# Supplementary material for: The kinesin-3 KIF1C undergoes liquid-liquid phase separation for accumulation of specific transcripts at the cell periphery
Source: EMBO J. 2024 Jun 19;43(15):3192–213. doi: 10.1038/s44318-024-00147-9 (PMC11294625; doi:10.1038/s44318-024-00147-9)
Supplement: Supplementary file 1 — Appendix [file 44318_2024_147_MOESM1_ESM.pdf]

Appendix

The kinesin-3 KIF1C undergoes liquid-liquid phase separation for accumulation of specific transcripts at the cell periphery

Qi Geng (耿奇)[1], Jakia Jannat Keya[2], Takashi Hotta[2], Kristen J Verhey[2 *]
1 Department of Molecular, Cellular, & Developmental Biology, University of Michigan, Ann Arbor, MI, USA;
2 Department of Cell & Developmental Biology, University of Michigan Medical School, Ann Arbor, MI, USA.
* Correspondence: kjverhey@umich.edu.

Table of contents

Appendix Figure S1. KIF1C does not colocalize with organelle markers. 2
Appendix Figure S2. Amino acid composition of KIF1C tail and IDR predictions of other kinesins. 3
Appendix Figure S3. The IDR is required for KIF1C punctum formation in cells. 4
Appendix Figure S4. Controls for experiments examining KIF1C LLPS behavior; KIF1C condensates do not enrich tubulin. 5
Appendix Figure S5. KIF1C enriches markers of RNA granules, KIF1Bα does not. 7
Appendix Figure S6. Controls for experiments showing KIF1C condensates behave like RNA granules; RNA binding proteins are enriched in KIF1C-bioID data. 8
Appendix Figure S7. Verification of KIF1C KO hTERT-RPE1 cells and controls for the smFISH experiments. 9
Appendix Figure S8. Protein purification and endogenous KIF1C concentration. 10

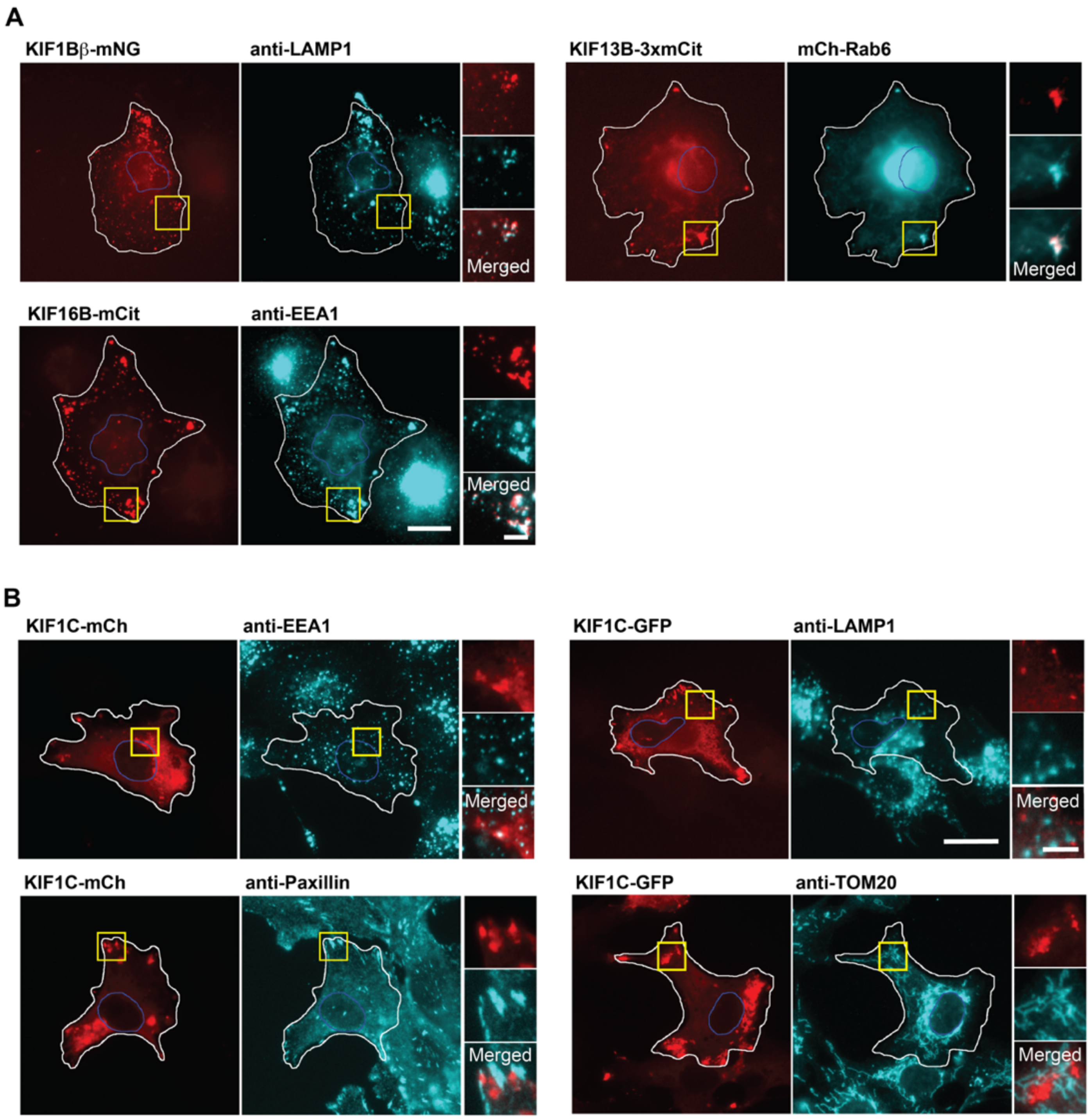

Appendix Figure S1. KIF1C does not colocalize with organelle markers.

(A) Representative images of COS-7 cells expressing fluorescently-tagged KIF1Bβ, KIF13B, or KIF16B. Rab6-positive vesicles are marked by mCherry-Rab6 co-transfection. Lysosomes are marked by anti-LAMP1 immunofluorescence. Early endosomes are marked by anti-EEA1 immunofluorescence. White lines indicate cell boundaries. Blue lines indicate nuclear boundaries. Yellow boxes indicate regions shown in magnified images to the right. Scale bar: 20 μm for whole cell views, 5 μm for magnified images.

(B) Immunofluorescence of markers for focal early endosomes (EEA1), lysosomes (LAMP1), adhesions (Paxillin), and mitochondria (TOM20) in hTERT-RPE1 cells expressing fluorescently-tagged KIF1C. Representative images are shown. White lines indicate cell boundaries. Blue lines indicate nuclear boundaries. Yellow boxes indicate regions shown in magnified images on the right. Scale bar: 20 μm for whole cell views, 5 μm for magnified images.

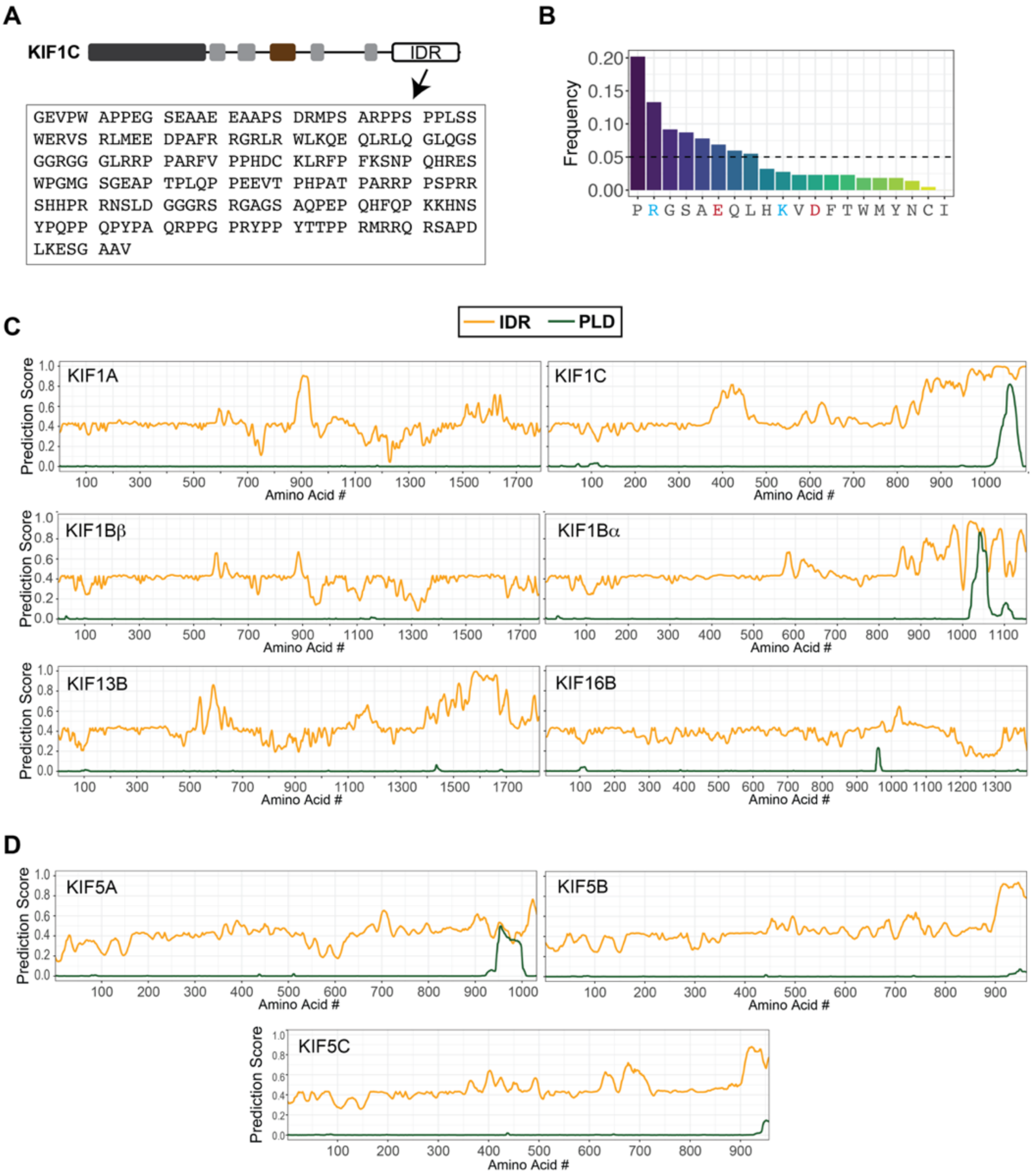

Appendix Figure S2. Amino acid composition of KIF1C tail and IDR predictions of other kinesins.

(A) The amino acid sequence of the KIF1C IDR.

(B) Frequency of amino acid residues in the KIF1C IDR. The horizontal dashed line indicates frequency equal to 0.05. On the x-axis, the positively-charged residues R and K are labelled blue; the negatively-charged residues E and D are labelled red.

(C,D) IUPred and PLAAC predictions of IDR and PLD, respectively, for (C) kinesin-3 family members and (D) kinesin-1 family members. x-axis: amino acid residue number; y-axis: predicted probability of the given residue being part of an IDR (orange line) or a PLD (green line).

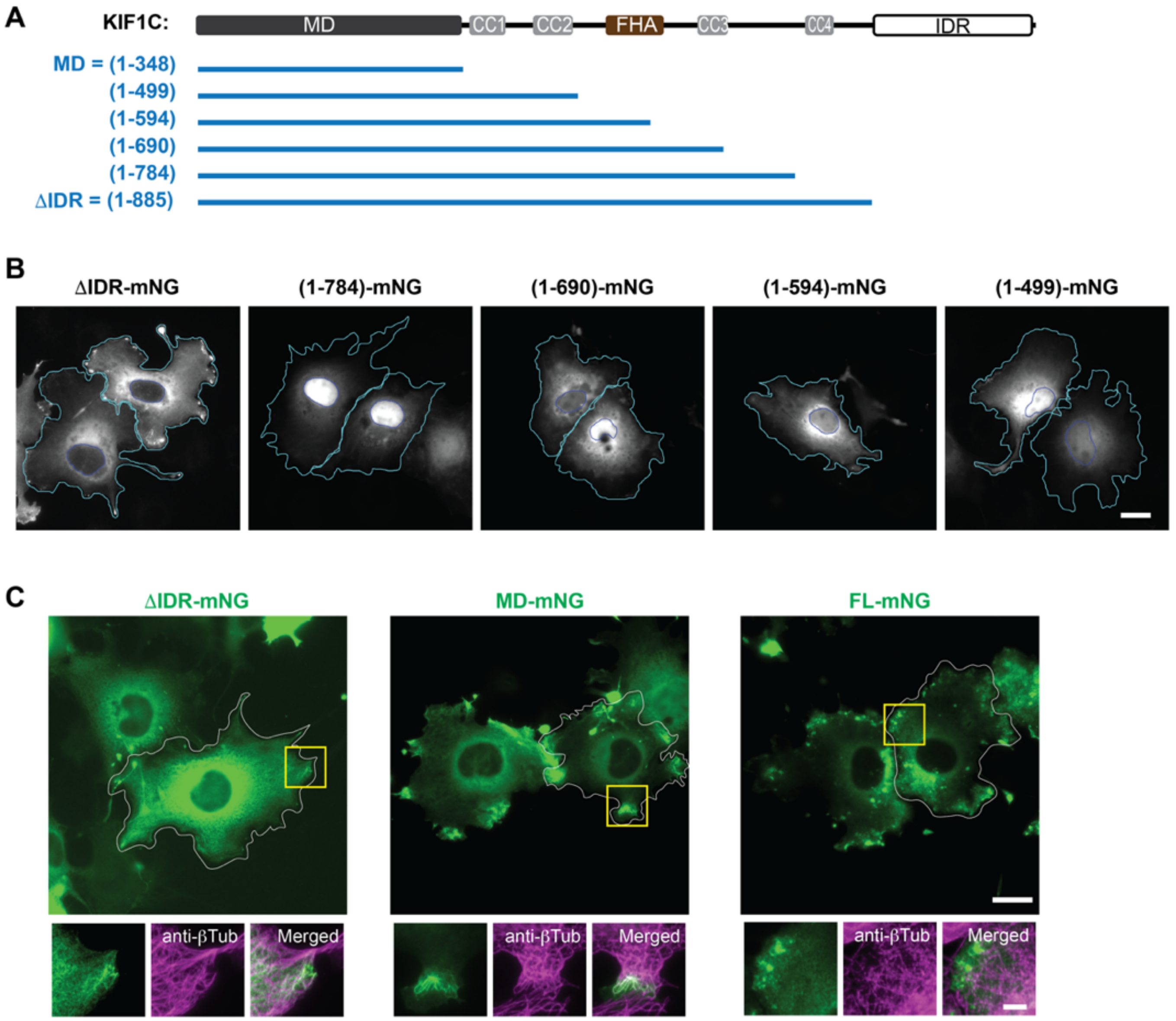

Appendix Figure S3. The IDR is required for KIF1C punctum formation in cells.

(A) Schematic of KIF1C serial truncations from the C-terminus.

(B) Representative images of KIF1C truncations in COS-7 cells. Scale bar: 20 μm. Cyan lines indicate cell boundaries. Blue lines indicate nuclear boundaries.

(C) Immunofluorescence of microtubules (anti-β-tubulin) in COS-7 cells expressing KIF1C(ΔIDR)-mNG, KIF1C(MD)-mNG, or KIF1C(FL)-mNG. Scale bar: 20 μm for whole cell views, 5 μm for magnified images.

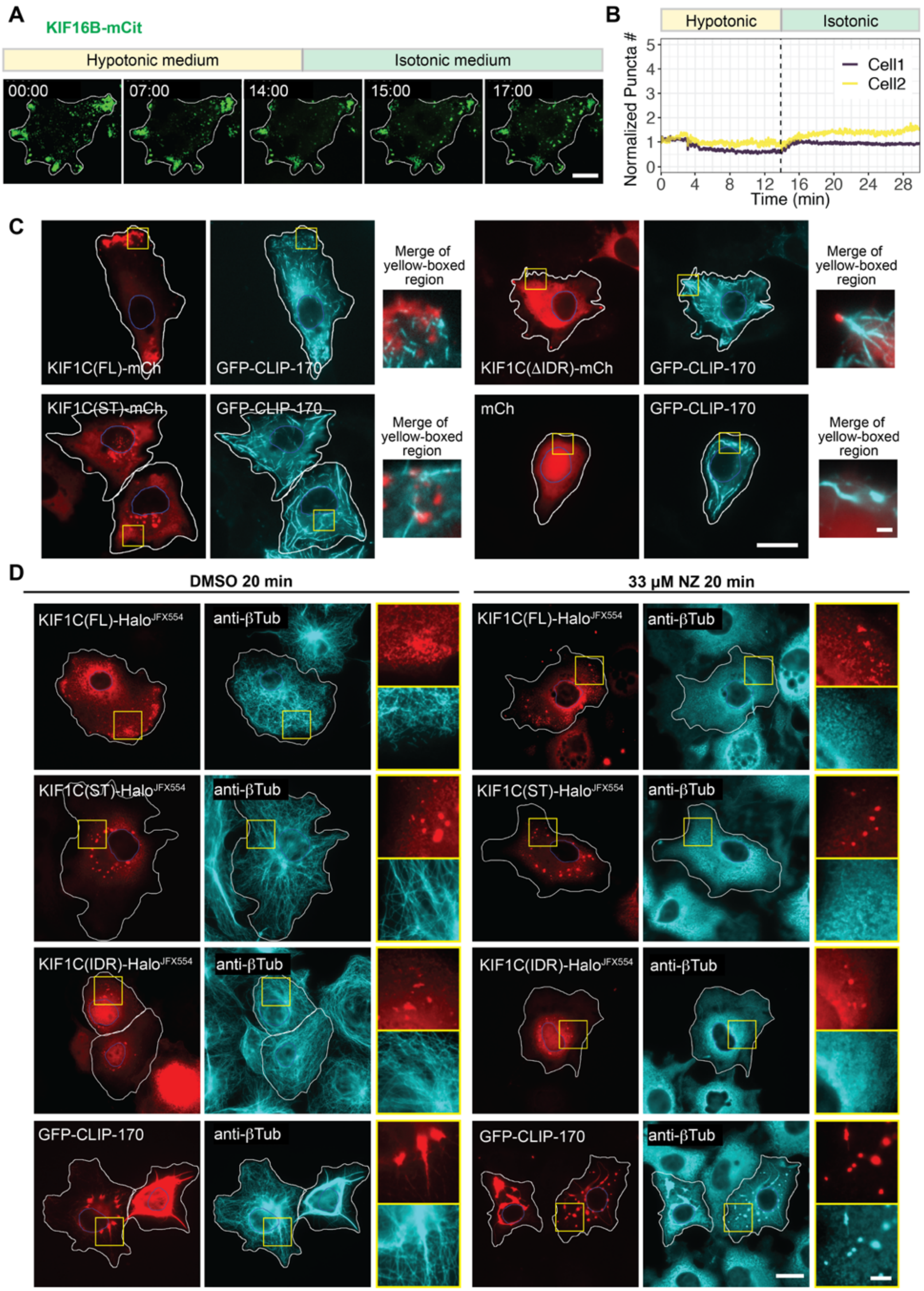

Appendix Figure S4. Controls for experiments examining KIF1C LLPS behavior; KIF1C condensates do not enrich tubulin.

(A,B) KIF16B puncta are not condensates (see Movie EV6). (A) Representative images of mCit-tagged KIF16B localization before treatment, during hypotonic treatment, and upon return to isotonic media. Scale bar: 20 μm. Time label is [min:sec]. (B) Quantification of change of puncta number over time in the cytoplasm dilution assay. x-axis: time, with a vertical dashed line indicating the time point of switching from the hypotonic media to the isotonic media. y-axis: puncta number or puncta area normalized against the first frame of live-cell imaging. The example cell in (A) is Cell1 in the plot.
(C,D) KIF1C condensates do not colocalize with CLIP-170 or tubulin. (C) Localization of mCh (control) or mCh-tagged KIF1C constructs (FL, ST, and ΔIDR) co-expressed with GFP-tagged CLIP-170 in hTERT-RPE1 cells. Representative images are shown. White lines indicate cell boundaries. Blue lines indicate nuclei boundaries. Yellow boxes indicate the regions displayed in the magnified images to the right. Scale bars: 20 μm for whole cell images, 2 μm for magnified images.
(D) Immunofluorescence of anti-βTubulin in COS-7 cells expressing HaloJFX^{554}-tagged KIF1C constructs (FL, ST, and IDR) or GFP-tagged CLIP-170. Cells were treated with (left) DMSO or (right) 33 μM nocodazole (NZ) for 20 min. White lines indicate cell boundaries. Yellow boxes indicate the regions displayed in the magnified images to the right. Scale bars: 20 μm for whole cell images, 5 μm for magnified images.

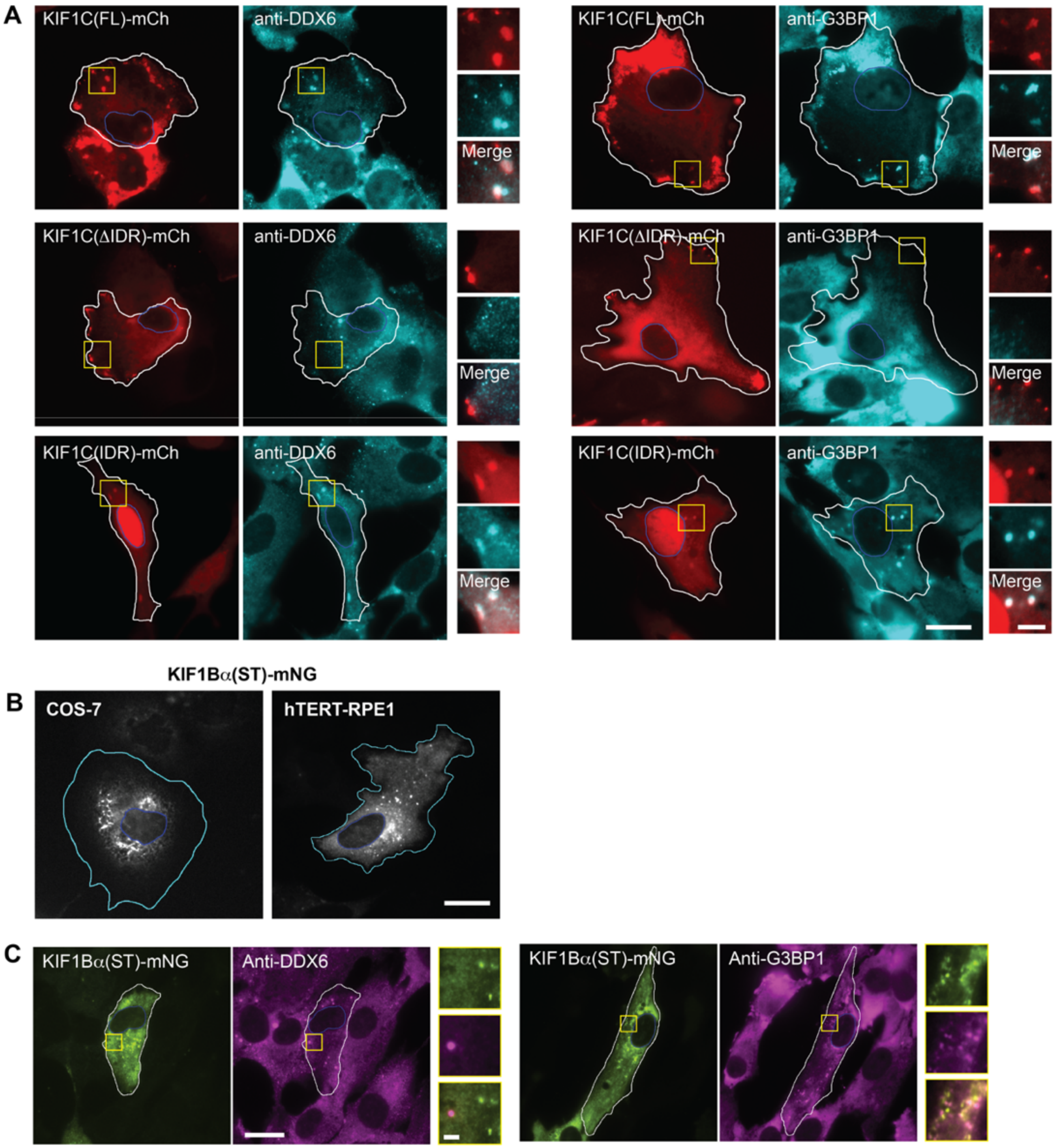

Appendix Figure S5. KIF1C enriches markers of RNA granules, KIF1Bα does not.

(A) Immunofluorescence for markers for P-bodies (anti-DDX6) and stress granules (anti-G3BP1) in hTERT-RPE1 cells expressing mCh-tagged KIF1C(FL), KIF1C(ΔIDR), or KIF1C(IDR). Representative images are shown. White lines indicate cell boundaries. Blue lines indicate nuclear boundaries. Yellow boxes indicate the regions displayed in the magnified images to the right. Scale bar: 20 µm for whole cell views, 5 µm for magnified images.

(B) Localization of KIF1Bα(ST)-mNG in COS-7 cells and hTERT-RPE1 cells. Representative images are shown. Cyan lines indicate cell boundaries. Scale bar: 20 µm.

(C) Immunofluorescence for markers for stress granules (anti-G3BP1) or P-bodies (anti-DDX6) in hTERT-RPE1 cells expressing KIF1Bα(ST)-mNG. Representative images are shown. White lines indicate cell boundaries. Yellow boxes indicate the regions displayed in the magnified images to the right. Scale bar: 20 µm for whole cell views, 5 µm for magnified images.

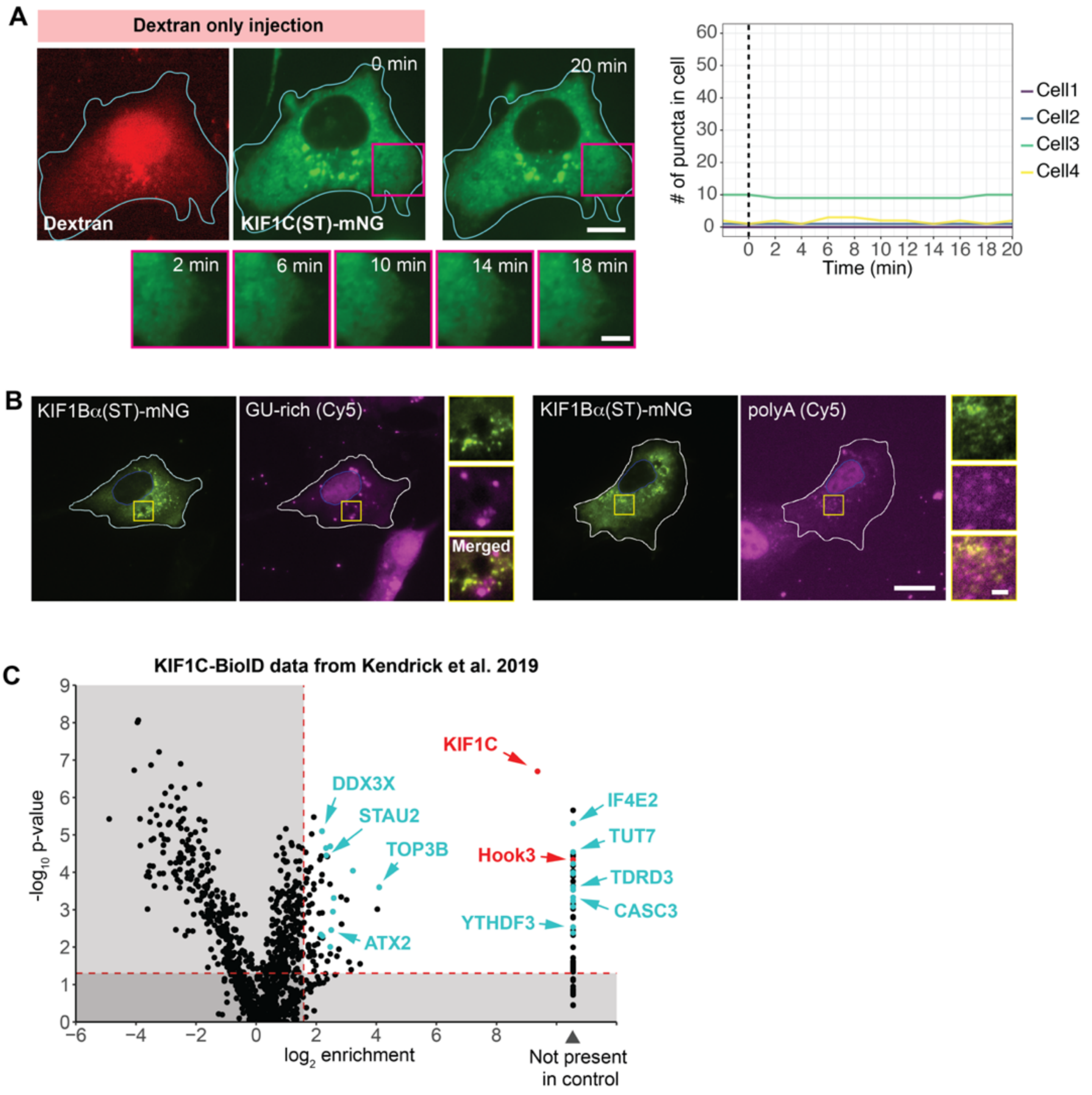

Appendix Figure S6. Controls for experiments showing KIF1C condensates behave like RNA granules; RNA binding proteins are enriched in KIF1C-bioID data.

(A) Representative images of a COS-7 cell expressing KIF1C(ST)-mNG before and after microinjection with fluorescent dextran control (see Movie EV8). Cyan lines indicate cell boundaries. Scale bar: 20 µm for whole cell views, 5 µm for magnified images. The graph on the right indicates quantification of the number of puncta in the magenta-boxed area over time. y-axis, number of KIF1C(ST) puncta. x-axis, time after injection. The vertical dashed lines represent the timepoint of microinjection.

(B) Representative images of Cy-5-labelled GU-rich or polyA RNA oligos introduced into hTERT-RPE1 cells expressing KIF1Bα(ST)-mNG. White lines indicate cell boundaries. Yellow boxes indicate the regions displayed in the magnified images to the right. Scale bars: 20 µm for whole cell views, 2 µm for magnified images.

(C) Volcano plot showing proteins identified in KIF1C-BioID experiments. Image modified from Kendrick et al., 2019, Fig. 1D. Proteins with an enrichment ratio > 3 and a p-value < 0.05 (two-tailed Student's t test) are included in the list. The interaction between KIF1C and Hook3 proteins (red text) has been characterized by Siddiqui et al., 2019 and Kendrick et al., 2019. The cyan spots indicated RBPs identified in the KIF1C interactome. The cyan text indicates several examples of RBPs involved in mRNA processing or decay. An interactive plot can be accessed at https://cdb-rshiny.med.umich.edu/Geng-KIF1C_bioID_visualization/.

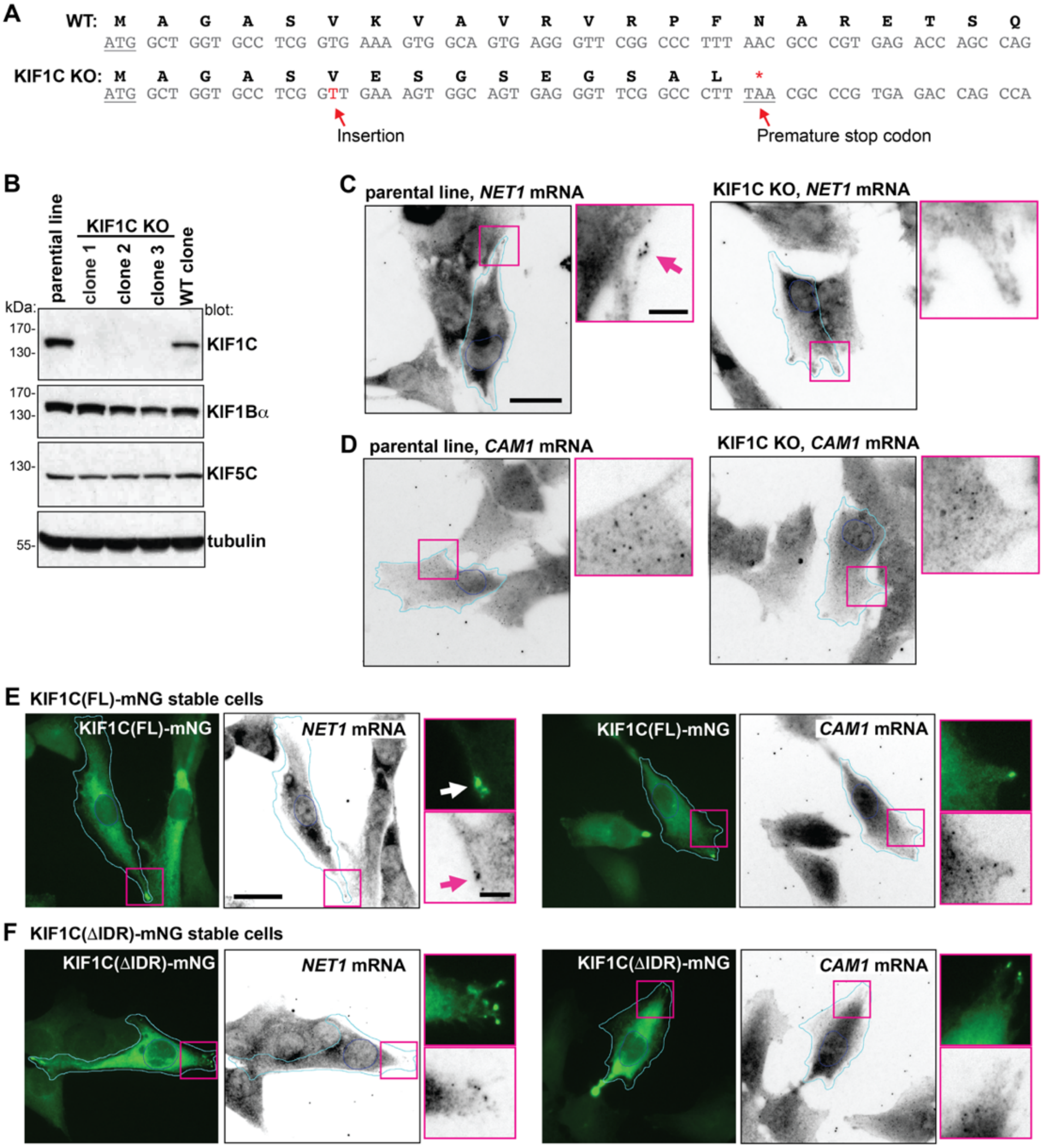

Appendix Figure S7. Verification of KIF1C KO hTERT-RPE1 cells and controls for the smFISH experiments.

(A) DNA sequences (gray text) obtained from WT (top) and KIF1C KO (bottom) hTERT-RPE1 cells and the resulting protein sequences (black text). The ATG start codon is underlined. An insertion of T (red text) in the sixth codon was found in all 3 clones of KIF1C KO cells, which results a premature stop codon (underlined and with a red asterisk).

(B) Western blot showing the absence of KIF1C protein in all three KIF1C KO clones, whereas the expression of kinesin-3 KIF1Bα and kinesin-1 KIF5C are unaffected.

(C-D) Representative smFISH images from 2 independent experiments showing the distribution of endogenous (C) *NET1* mRNA and (D) *CAM1* mRNA in WT parental cells and KIF1C KO cells. The magenta arrow indicates a cell protrusion with *NET1* mRNA enrichment. Scale bar: 20 µm for whole cell views, 5 µm for magnified images.

(E-F) Representative smFISH images from 2 independent experiments showing the distribution of endogenous *NET1* mRNA and *CAM1* mRNA in (E) KIF1C(FL)-mNG and (F) KIF1C(ΔIDR)-mNG stable cells generated from KIF1C KO cells. Cyan lines indicate cell boundaries. Magenta boxes indicate the regions displayed in the magnified images to the right. The arrows indicate a cell protrusion with KIF1C(FL)-mNG protein enrichment (white) and *NET1* mRNA enrichment (magenta). Scale bar: 20 µm for whole cell views, 5 µm for magnified images.

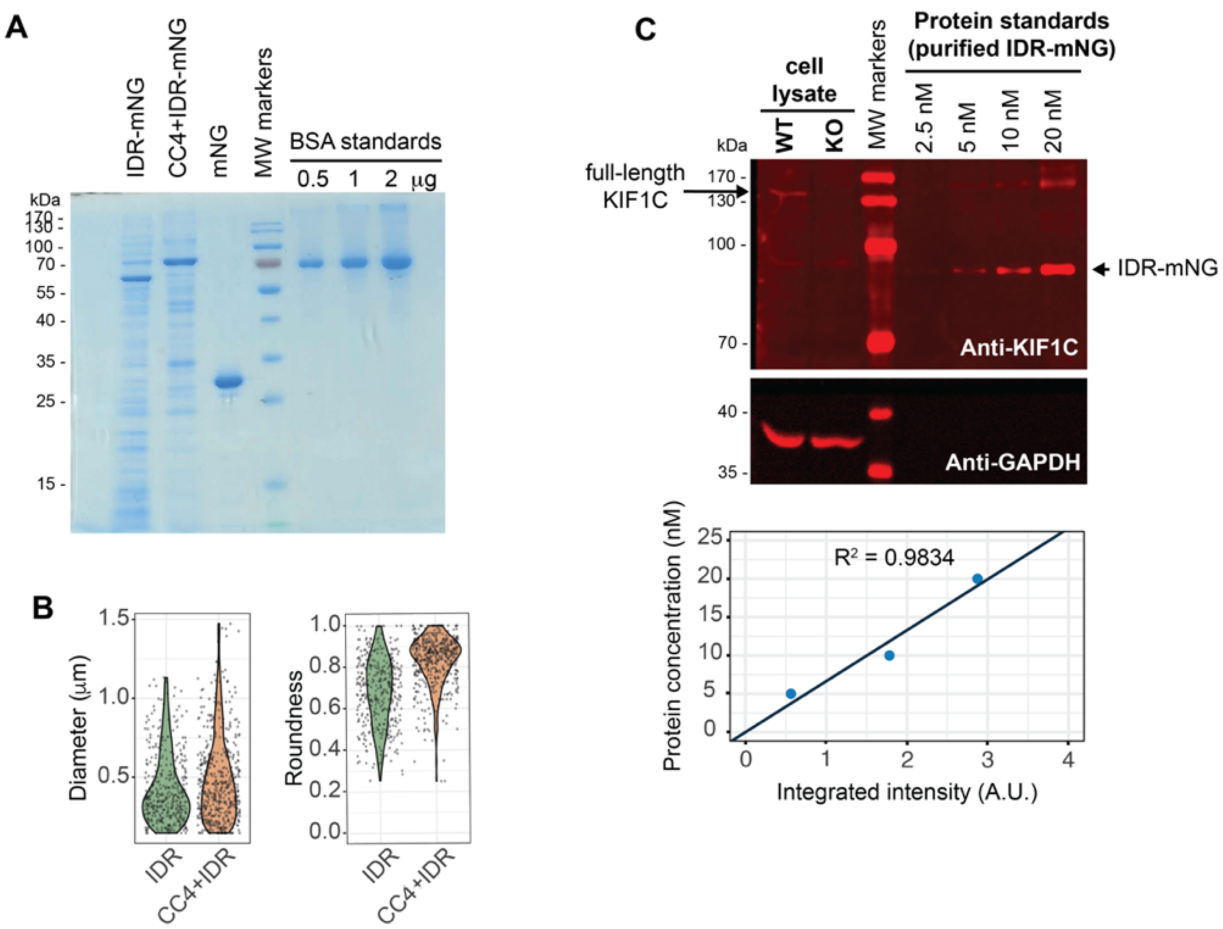

Appendix Figure S8. Protein purification and endogenous KIF1C concentration.

(A) Purified KIF1C(IDR)-mNG, KIF1C(CC4+IDR)-mNG, and mNG separated by SDS-PAGE. Bovine serum albumin (BSA) was run as protein standards.

(B) Quantification of condensate size (diameter) and shape (roundness) in 100 mM NaCl buffer. Punctum diameter (mean ± SD): 0.43 ± 0.22 μm for IDR-mNG and 0.48 ± 0.27 μm for CC4+IDR-mNG , p-value = 0.0012 (t-test). Roundness (mean ± SD): 0.69 ± 0.16 μm for IDR-mNG and 0.83 ± 0.12 μm for CC4+IDR-mNG, p-value < 0.001 (t-test). N=389 puncta for IDR-mNG; N=503 puncta for CC4+IDR-mNG.

(C) Estimation of the endogenous KIF1C concentration in hTERT-RPE cells by western blot. Cell lysates of WT and KIF1C KO hTERT-RPE1 cells were probed by western blotting with an antibody against KIF1C and an antibody against GAPDH (loading control). Purified KIF1C(IDR)-mNG was used as protein standards to generate a standard curve (bottom) for calculating the concentration of endogenous KIF1C.
