# Supplementary figures and images for: The kinesin-3 KIF1C undergoes liquid-liquid phase separation for accumulation of specific transcripts at the cell periphery
Source: EMBO J. 2024 Jun 19;43(15):3192–213. doi: 10.1038/s44318-024-00147-9 (PMC11294625; doi:10.1038/s44318-024-00147-9)

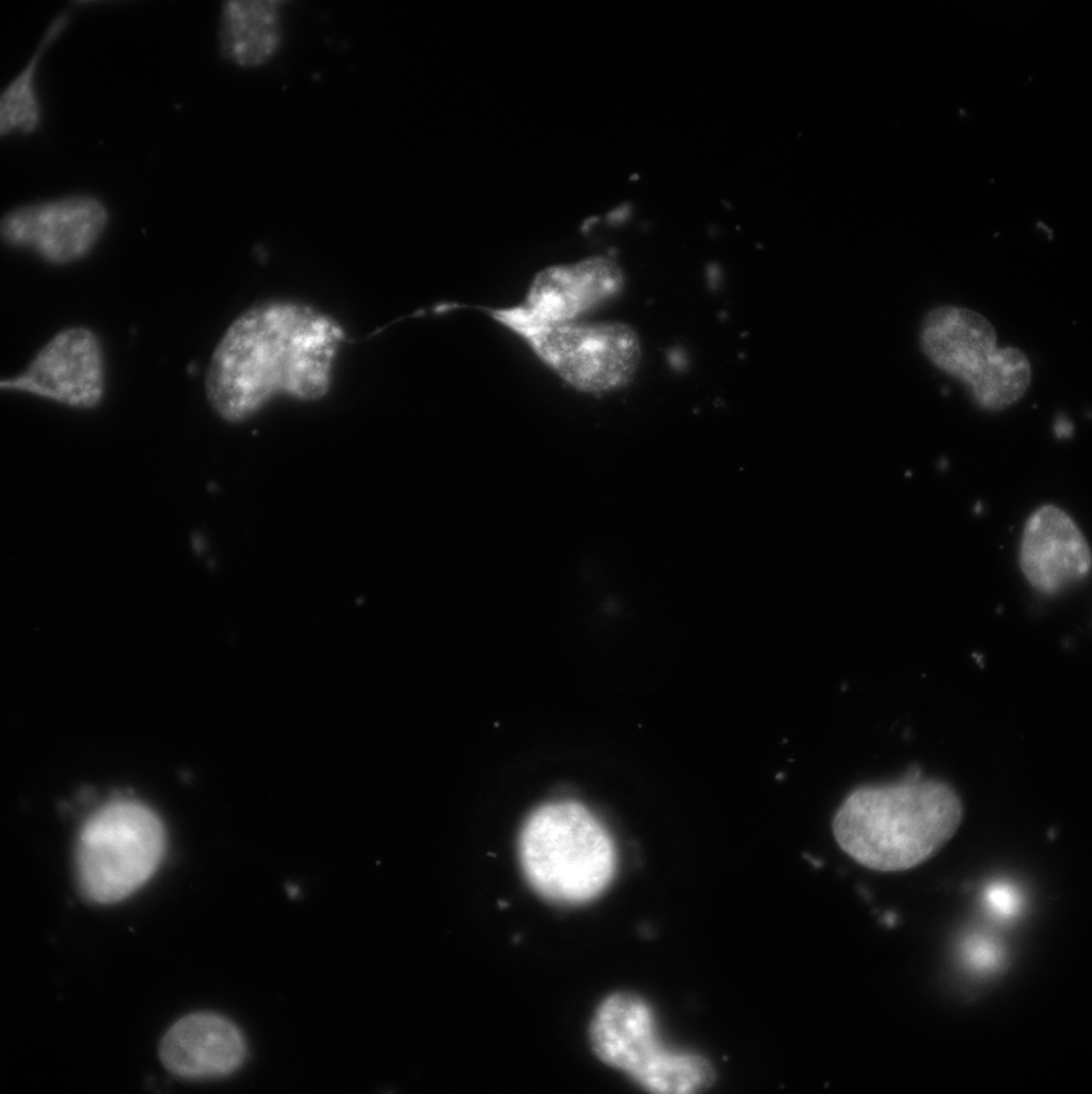

Supplement: Supplementary file 13 — Source data Fig. 1 [file 44318_2024_147_MOESM13_ESM.zip › Figure 1/1B/KIF16B_mCit_transfection_COS7.tif]

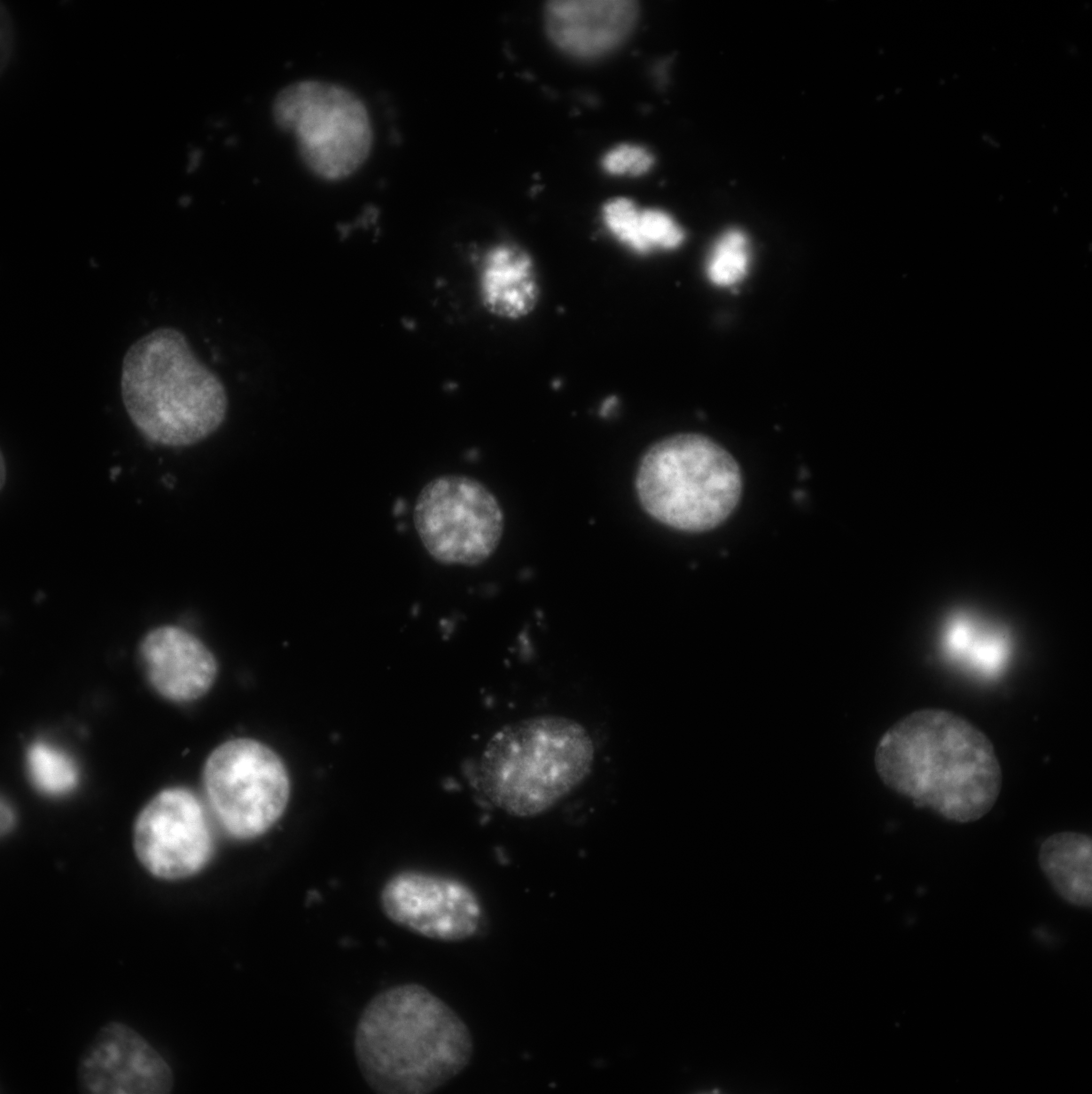

Supplement: Supplementary file 13 — Source data Fig. 1 [file 44318_2024_147_MOESM13_ESM.zip › Figure 1/1B/KIF1C_GFP_transfection_COS7.tif]

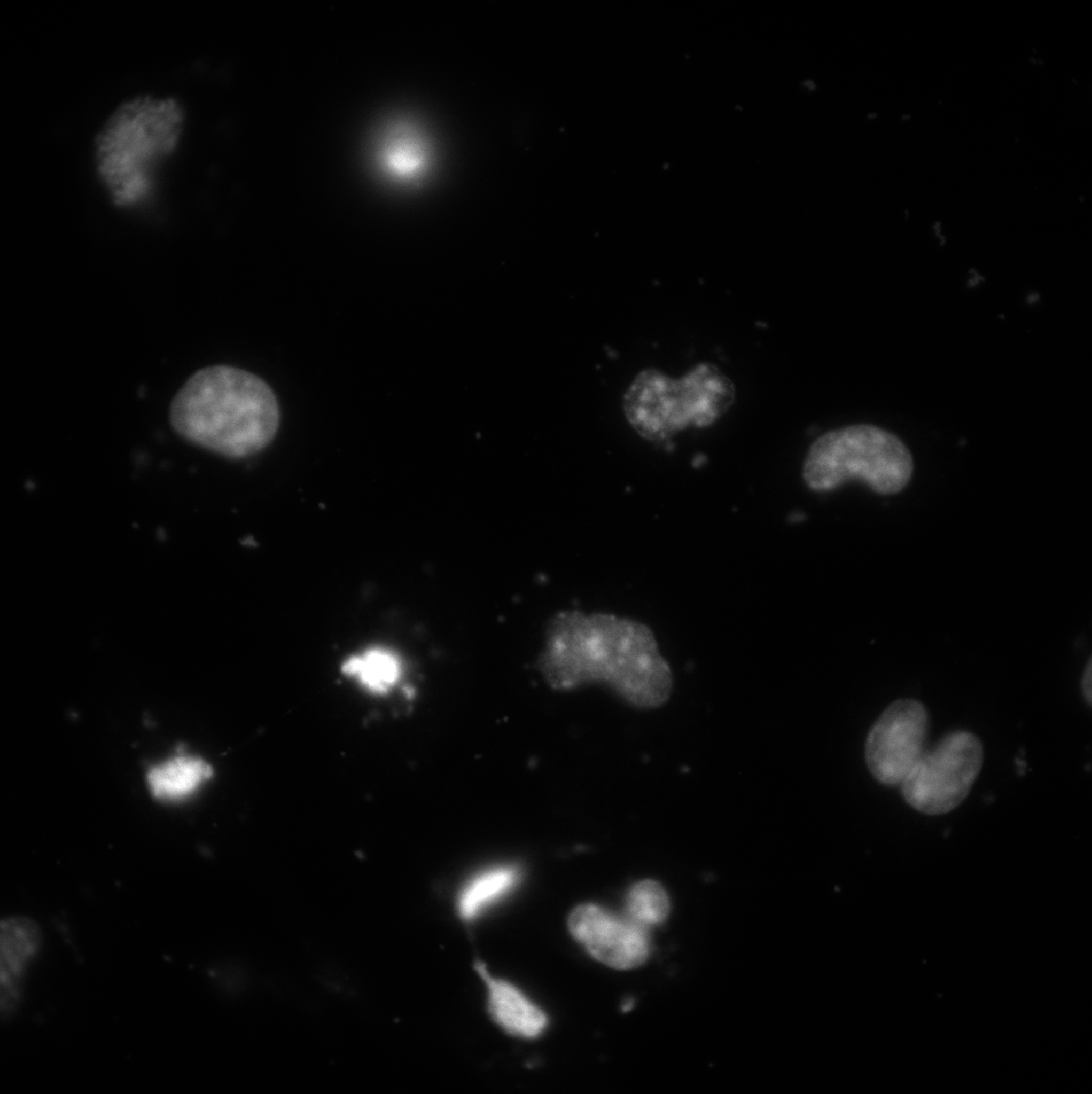

Supplement: Supplementary file 13 — Source data Fig. 1 [file 44318_2024_147_MOESM13_ESM.zip › Figure 1/1B/KIF1Bbeta_mNG_transfection_COS7.tif]

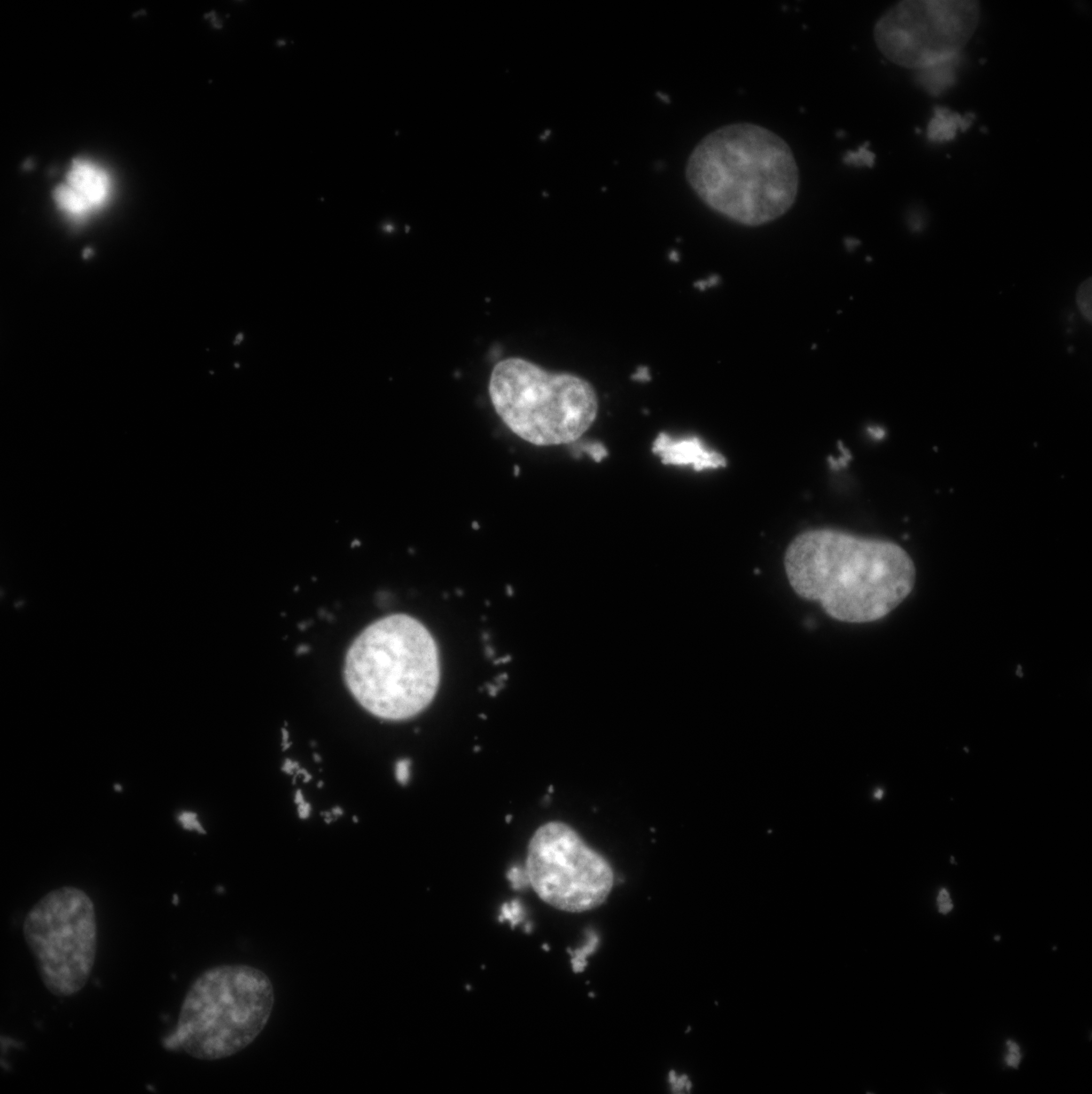

Supplement: Supplementary file 13 — Source data Fig. 1 [file 44318_2024_147_MOESM13_ESM.zip › Figure 1/1B/KIF13B_3xmCit_transfection_COS7.tif]

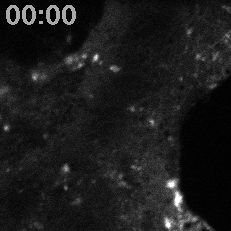

Supplement: Supplementary file 13 — Source data Fig. 1 [file 44318_2024_147_MOESM13_ESM.zip › Figure 1/1D/KIF1C_GFP_transfected_COS7_30umx30um.tif]

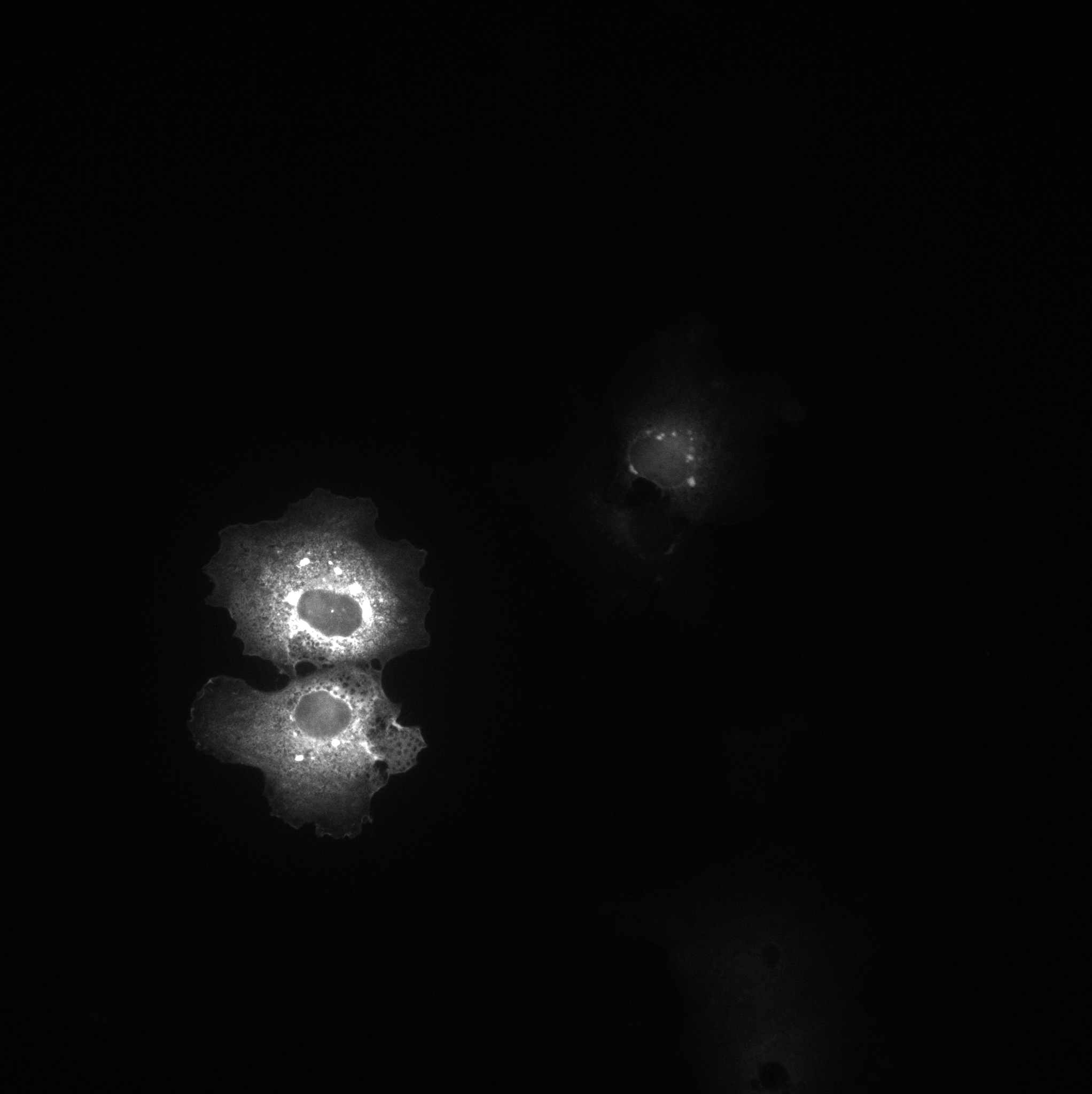

Supplement: Supplementary file 14 — Source data Fig. 2 [file 44318_2024_147_MOESM14_ESM.zip › Figure 2/2C/CC4+IDR_mNG_COS7.tif]

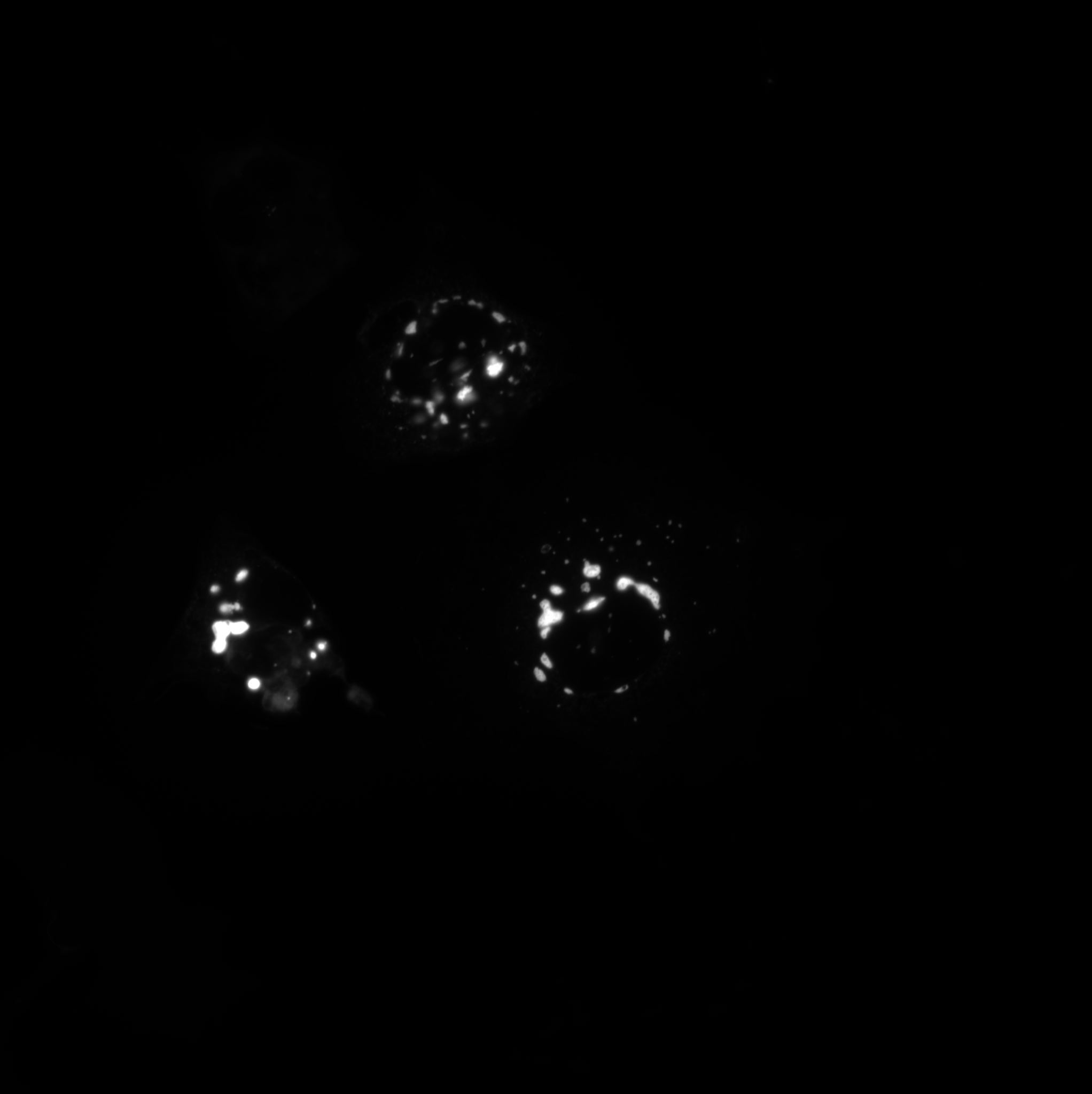

Supplement: Supplementary file 14 — Source data Fig. 2 [file 44318_2024_147_MOESM14_ESM.zip › Figure 2/2C/ST_mNG_COS7.tif]

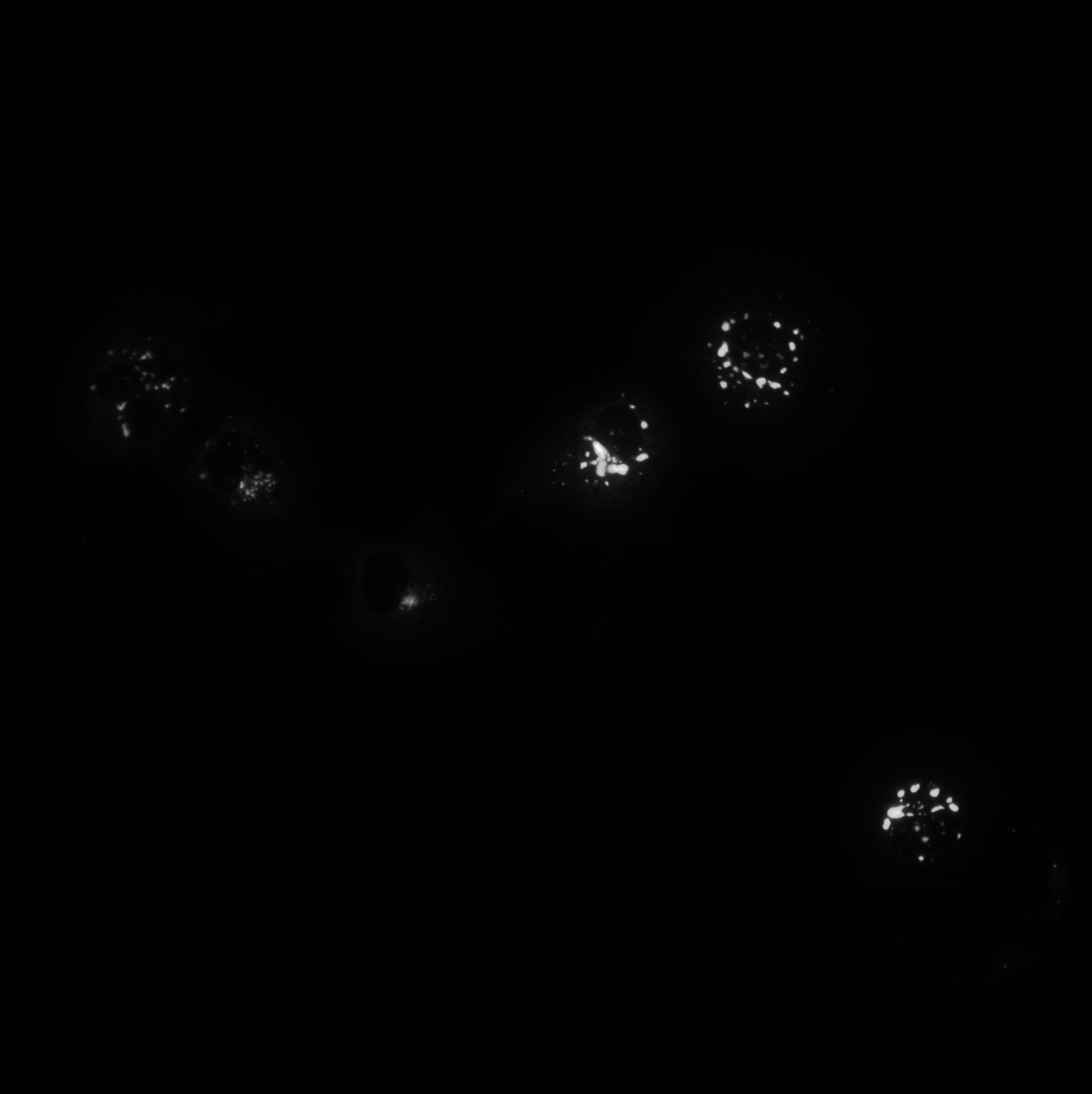

Supplement: Supplementary file 14 — Source data Fig. 2 [file 44318_2024_147_MOESM14_ESM.zip › Figure 2/2C/500t1103_mNG_COS7.tif]

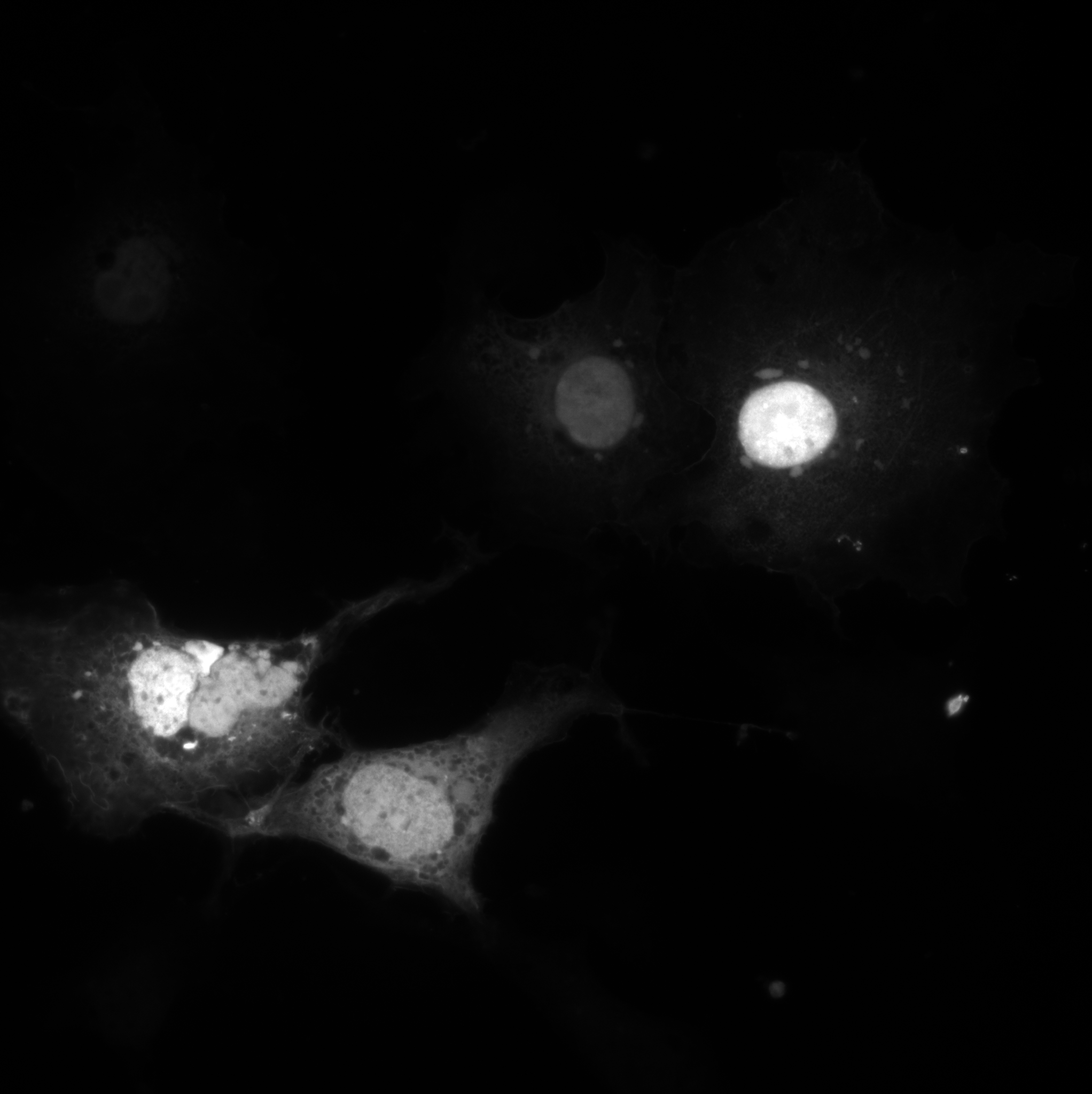

Supplement: Supplementary file 14 — Source data Fig. 2 [file 44318_2024_147_MOESM14_ESM.zip › Figure 2/2C/IDR_mNG_COS7.tif]

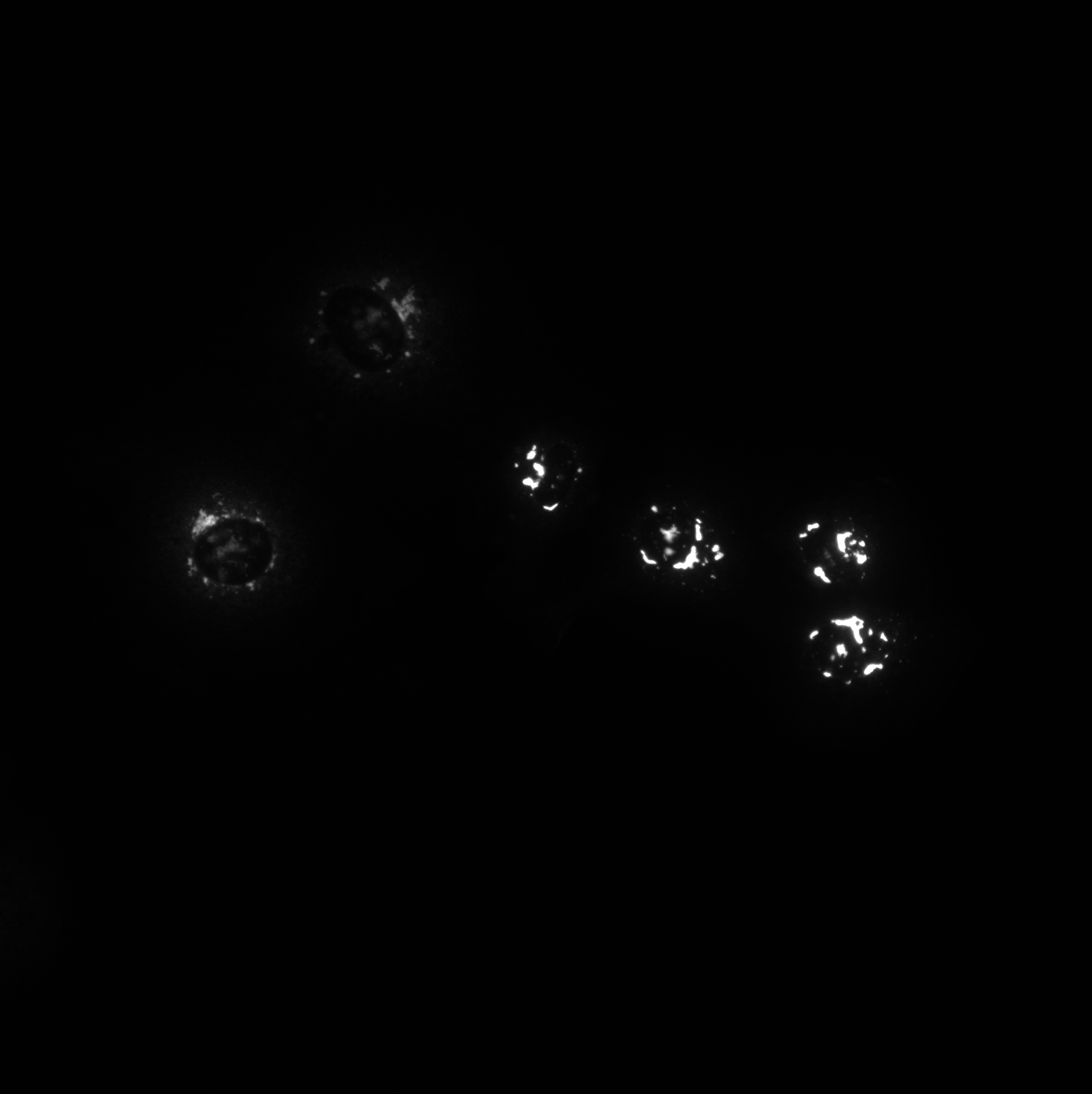

Supplement: Supplementary file 14 — Source data Fig. 2 [file 44318_2024_147_MOESM14_ESM.zip › Figure 2/2C/691t1103_mNG_COS7.tif]

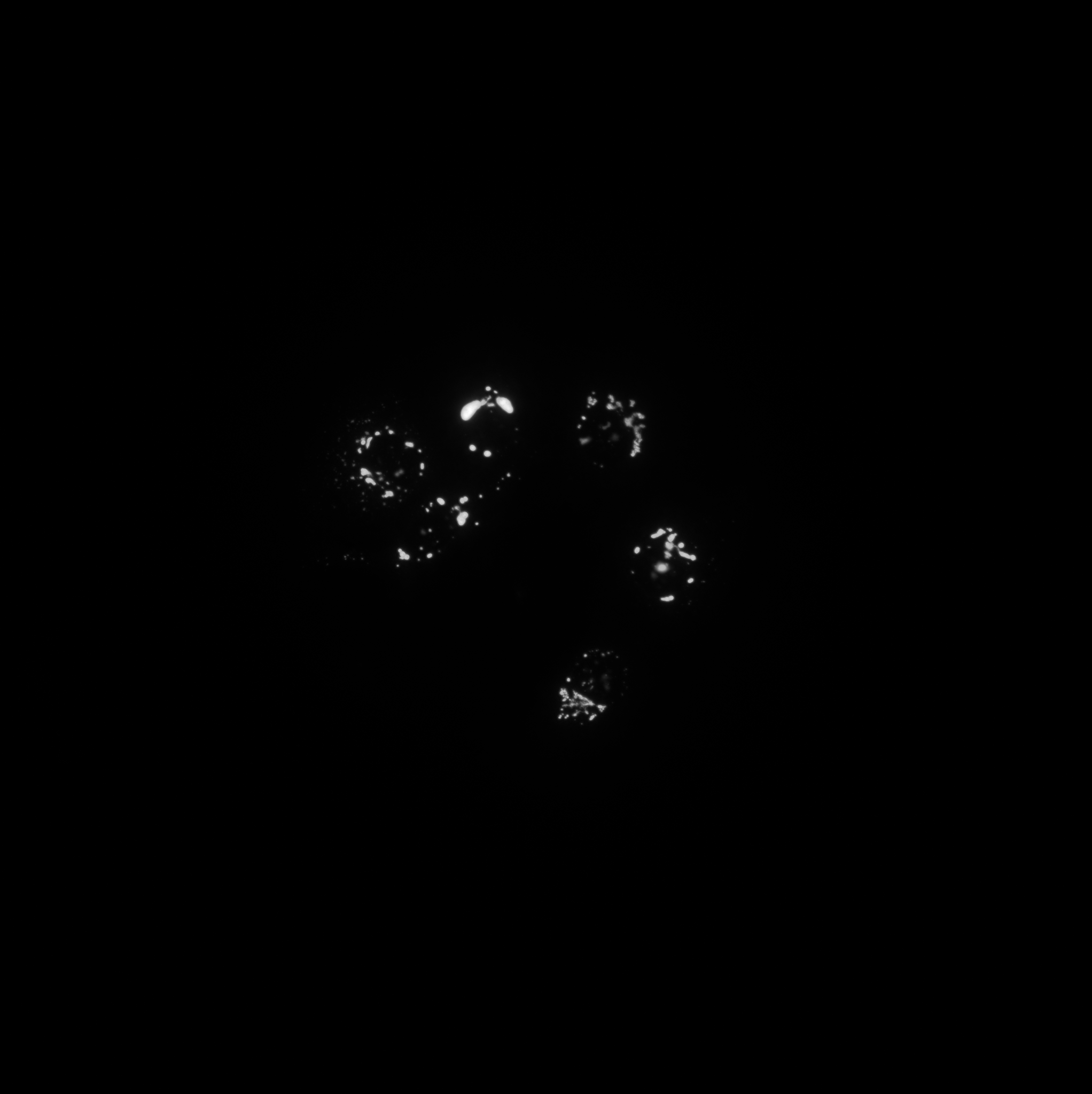

Supplement: Supplementary file 14 — Source data Fig. 2 [file 44318_2024_147_MOESM14_ESM.zip › Figure 2/2C/595t1103_mNG_COS7.tif]

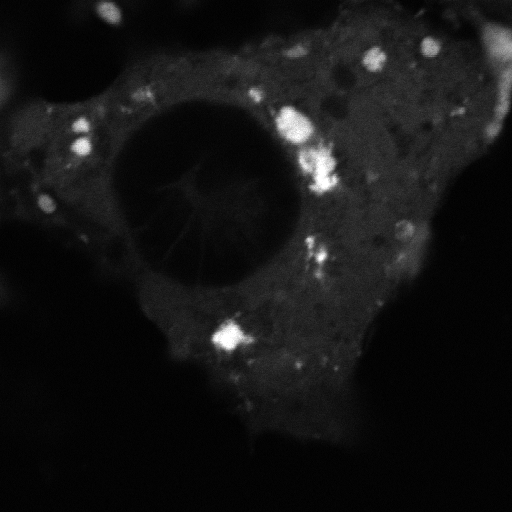

Supplement: Supplementary file 15 — Source data Fig. 3 [file 44318_2024_147_MOESM15_ESM.zip › Figure 3/3B/KIF1CFLmCh_mCh_channel.tif]

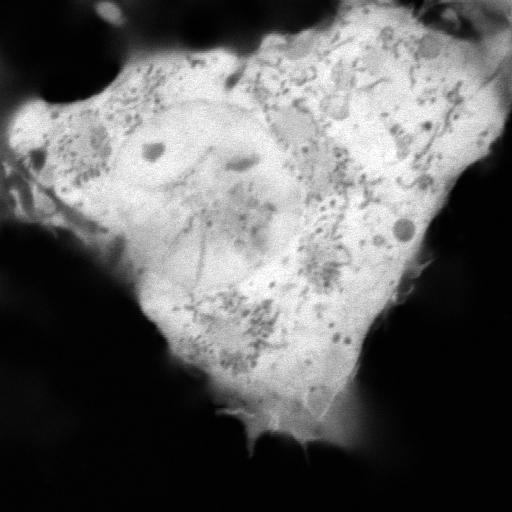

Supplement: Supplementary file 15 — Source data Fig. 3 [file 44318_2024_147_MOESM15_ESM.zip › Figure 3/3B/KIF1CFLmCh_GFP_channel.tif]

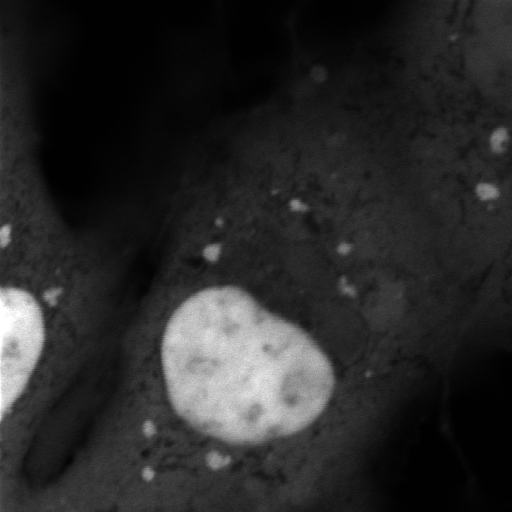

Supplement: Supplementary file 15 — Source data Fig. 3 [file 44318_2024_147_MOESM15_ESM.zip › Figure 3/3B/KIF1CIDRmCh_mCh_channel.tif]

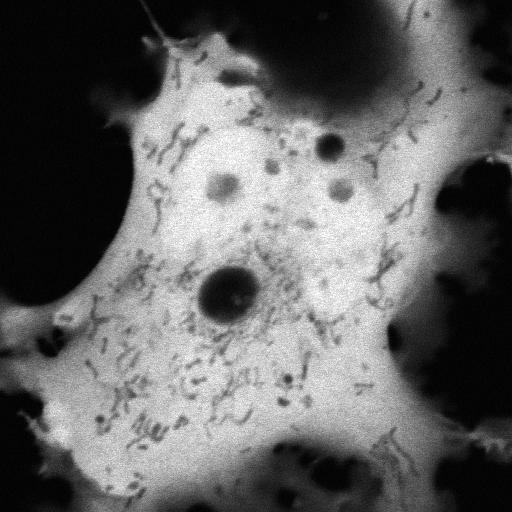

Supplement: Supplementary file 15 — Source data Fig. 3 [file 44318_2024_147_MOESM15_ESM.zip › Figure 3/3B/KIF1CSTmCh_GFP_channel.tif]

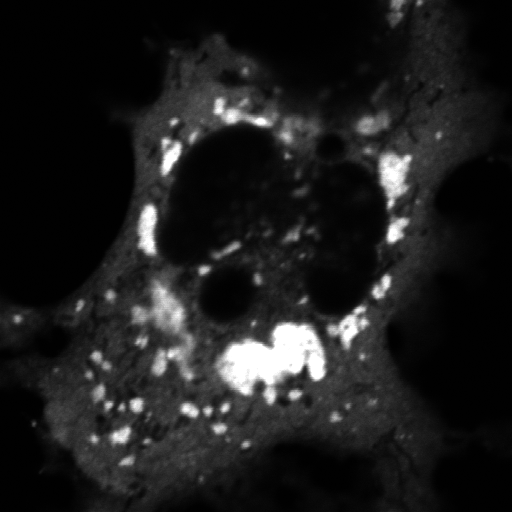

Supplement: Supplementary file 15 — Source data Fig. 3 [file 44318_2024_147_MOESM15_ESM.zip › Figure 3/3B/KIF1CSTmCh_mCh_channel.tif]

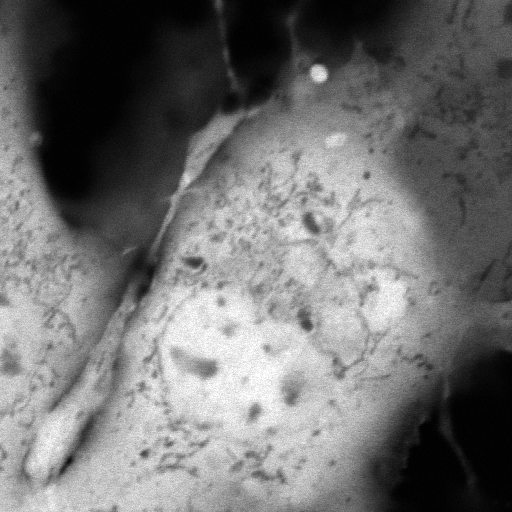

Supplement: Supplementary file 15 — Source data Fig. 3 [file 44318_2024_147_MOESM15_ESM.zip › Figure 3/3B/KIF1CIDRmCh_GFP_channel.tif]

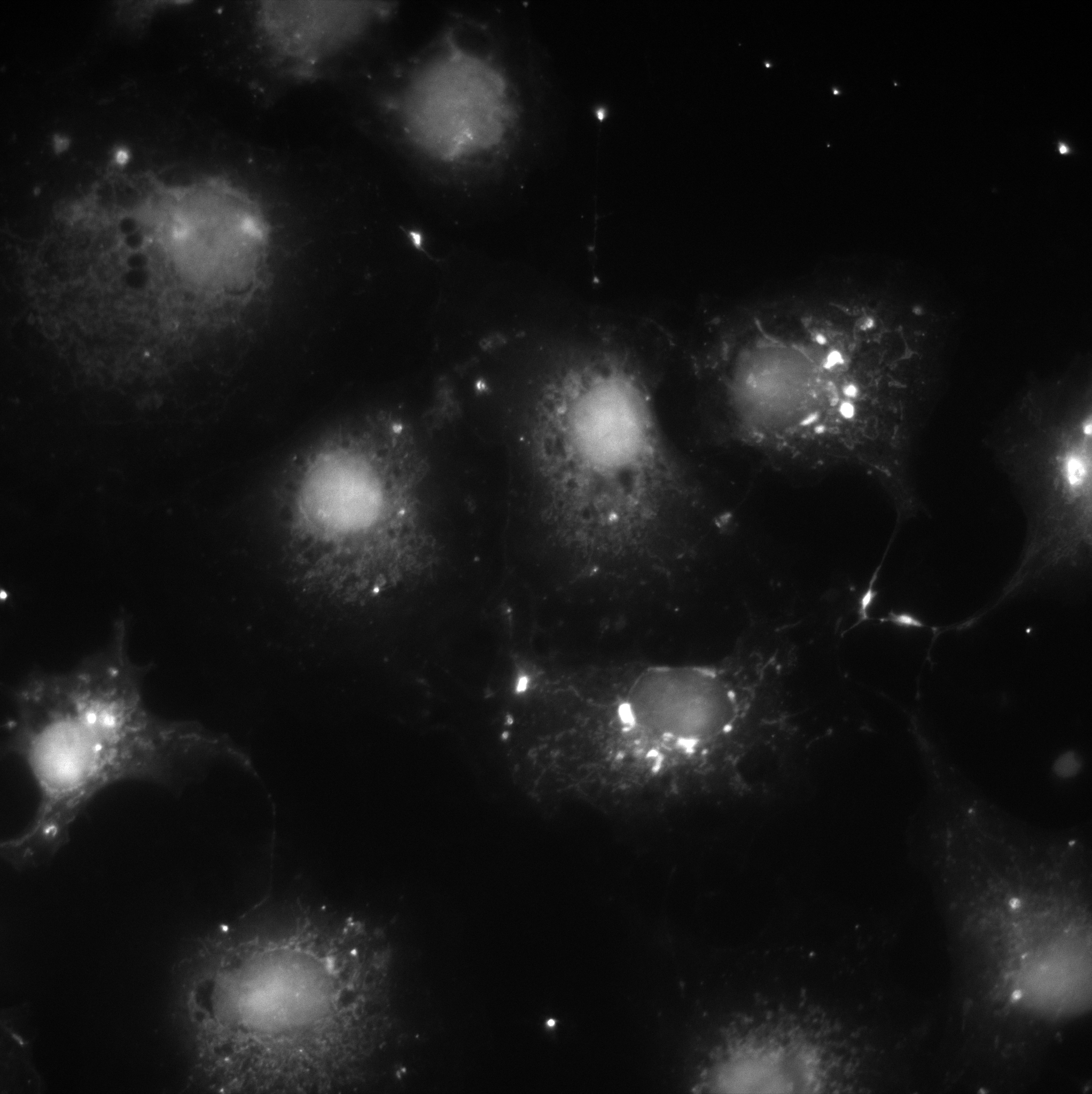

Supplement: Supplementary file 16 — Source data Fig. 4 [file 44318_2024_147_MOESM16_ESM.zip › Figure 4/4B/COS7_KIF1CST_4hr_transfection_GU_20uM.tif]

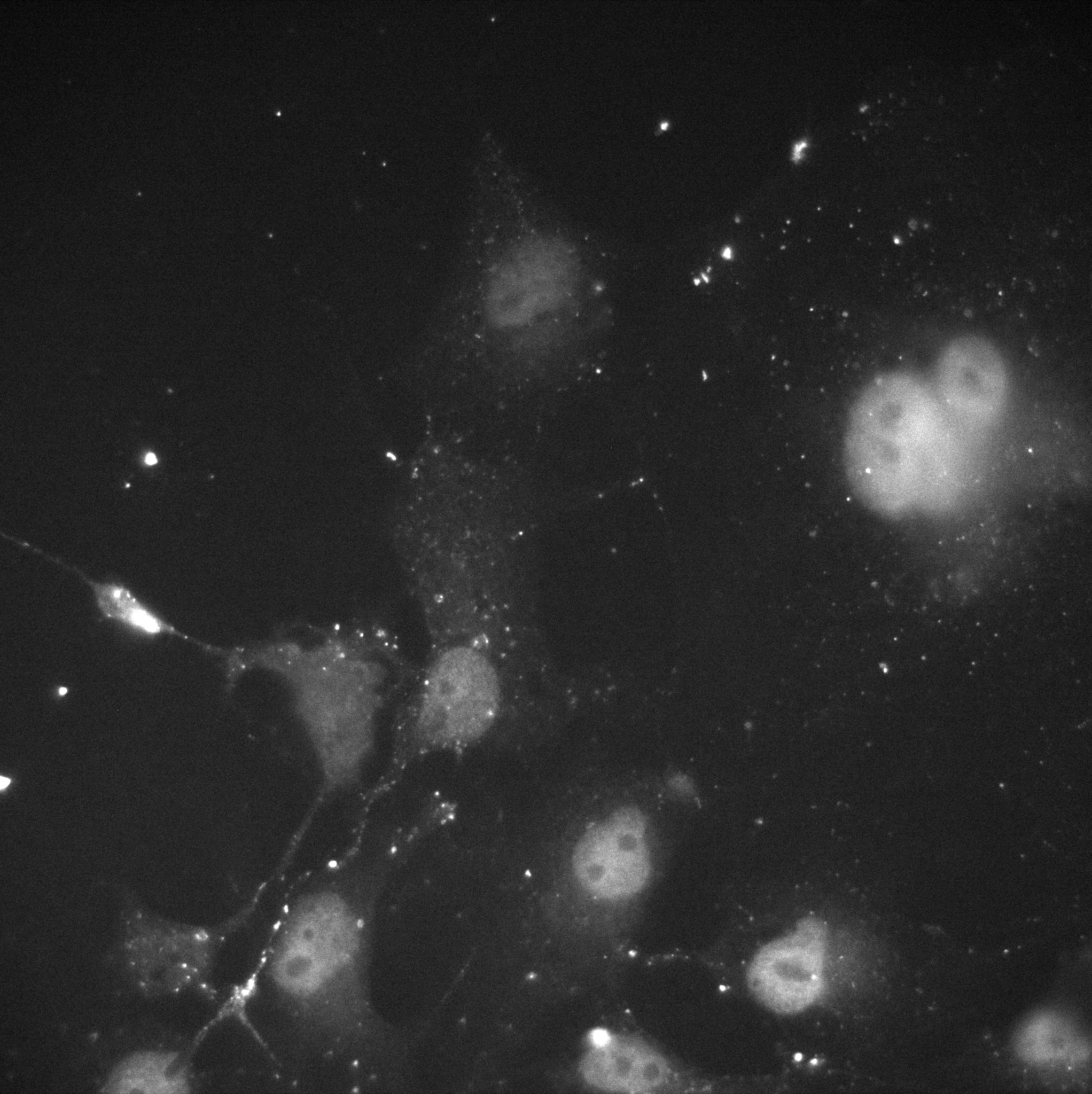

Supplement: Supplementary file 16 — Source data Fig. 4 [file 44318_2024_147_MOESM16_ESM.zip › Figure 4/4B/COS7_KIF1CIDR_4hr_transfection_A13_20uM.tif]

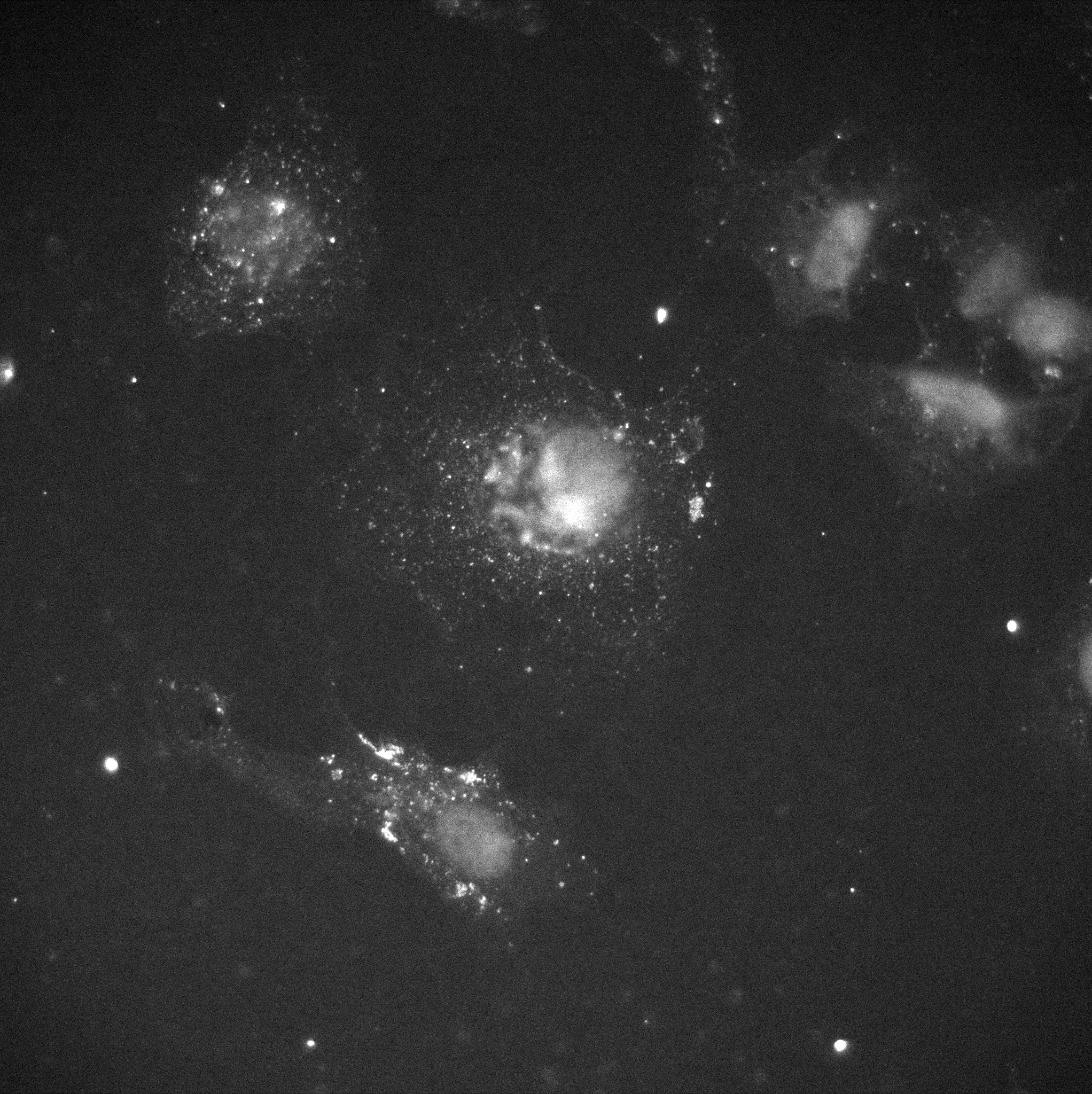

Supplement: Supplementary file 16 — Source data Fig. 4 [file 44318_2024_147_MOESM16_ESM.zip › Figure 4/4B/COS7_KIF1CST_4hr_transfection_A13_20uM.tif]

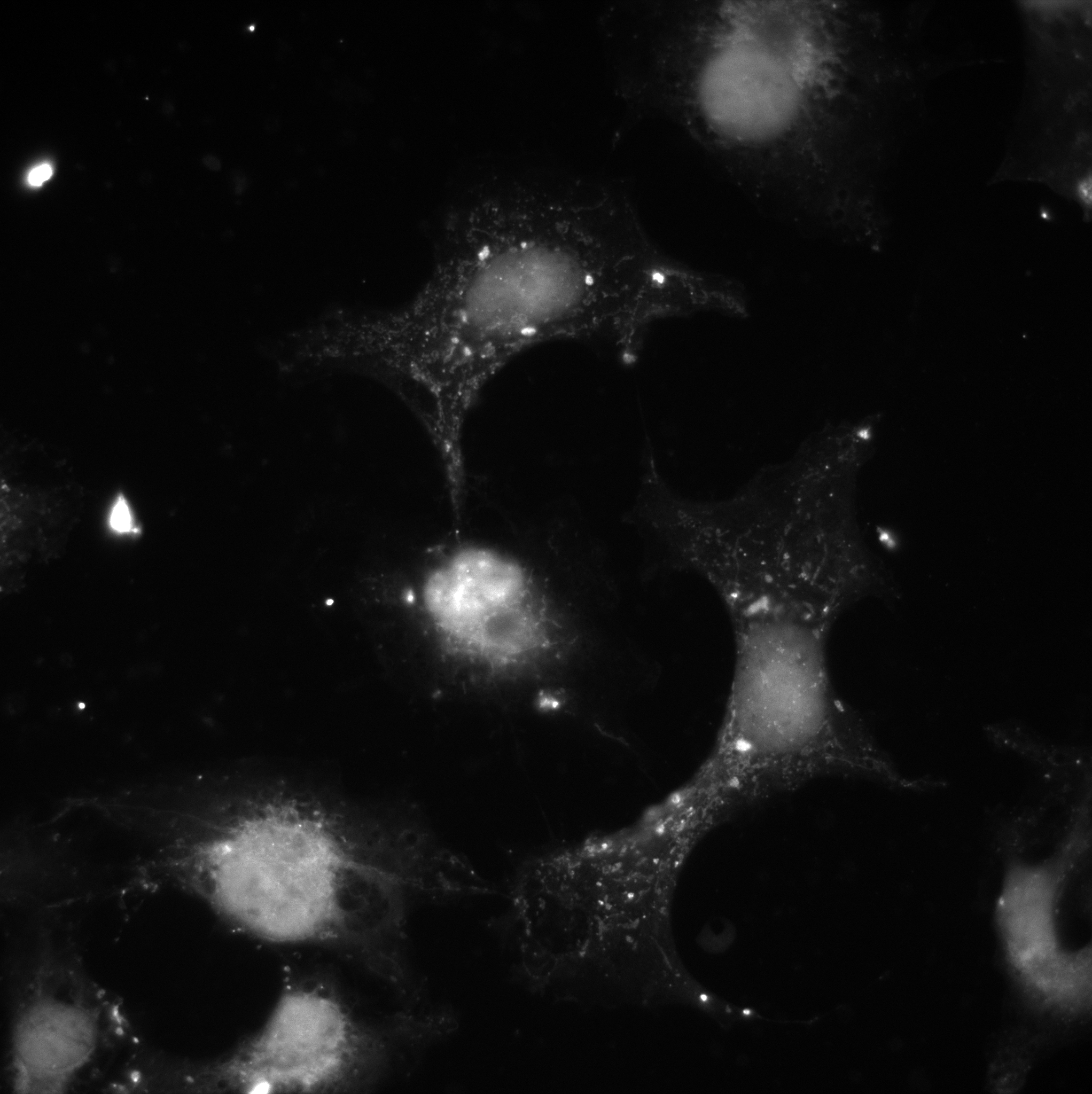

Supplement: Supplementary file 16 — Source data Fig. 4 [file 44318_2024_147_MOESM16_ESM.zip › Figure 4/4B/COS7_KIF1CIDR_4hr_transfection_GU_20uM.tif]

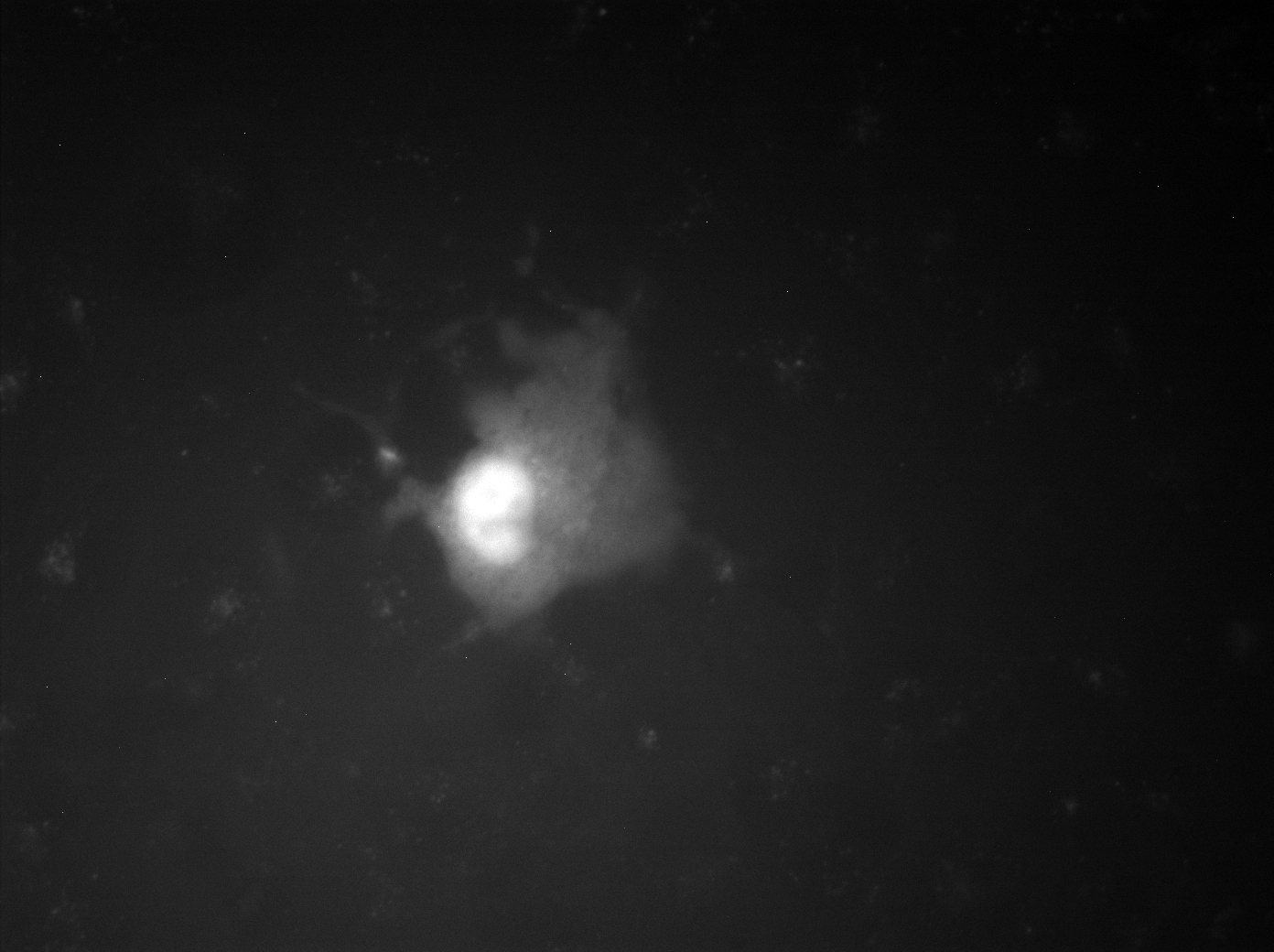

Supplement: Supplementary file 16 — Source data Fig. 4 [file 44318_2024_147_MOESM16_ESM.zip › Figure 4/4A/cell_3 after RNaseA_injection_red_channel.tif]

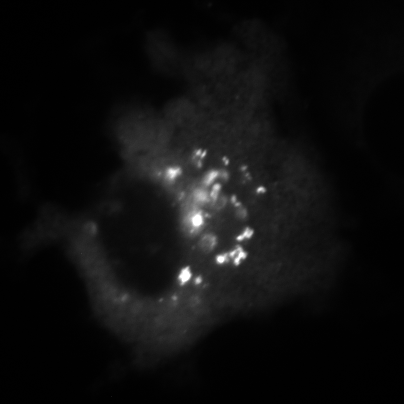

Supplement: Supplementary file 16 — Source data Fig. 4 [file 44318_2024_147_MOESM16_ESM.zip › Figure 4/4A/cell_3 after RNaseA_injection_100x100um.tif]

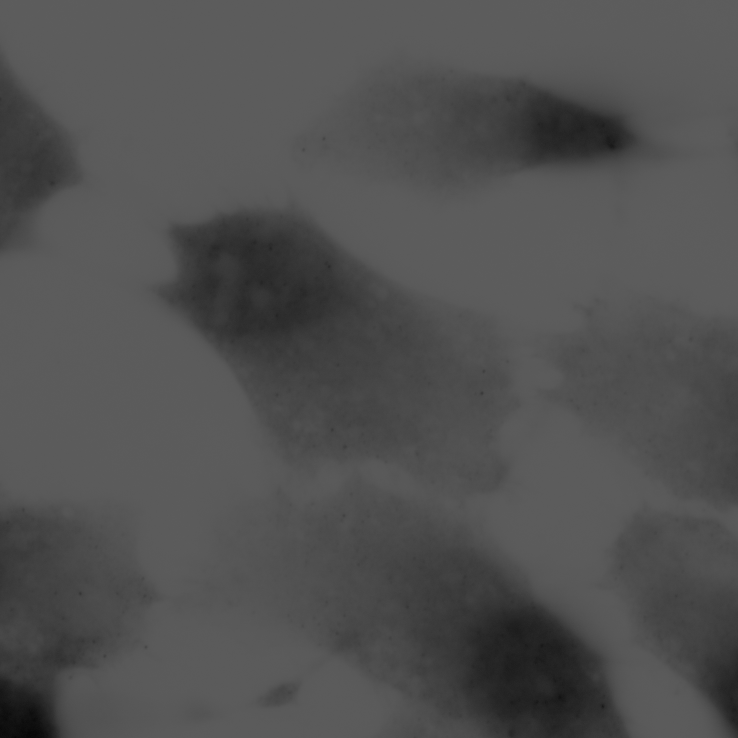

Supplement: Supplementary file 17 — Source data Fig. 5 [file 44318_2024_147_MOESM17_ESM.zip › Figure 5/5A/KIF1CKO_clone1_RPE.tif]

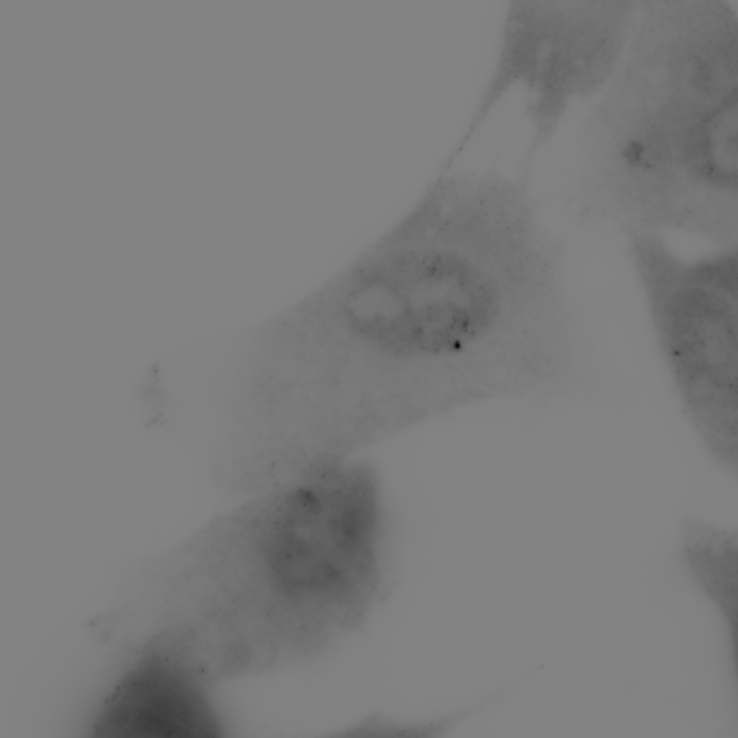

Supplement: Supplementary file 17 — Source data Fig. 5 [file 44318_2024_147_MOESM17_ESM.zip › Figure 5/5A/WT_RPE.tif]

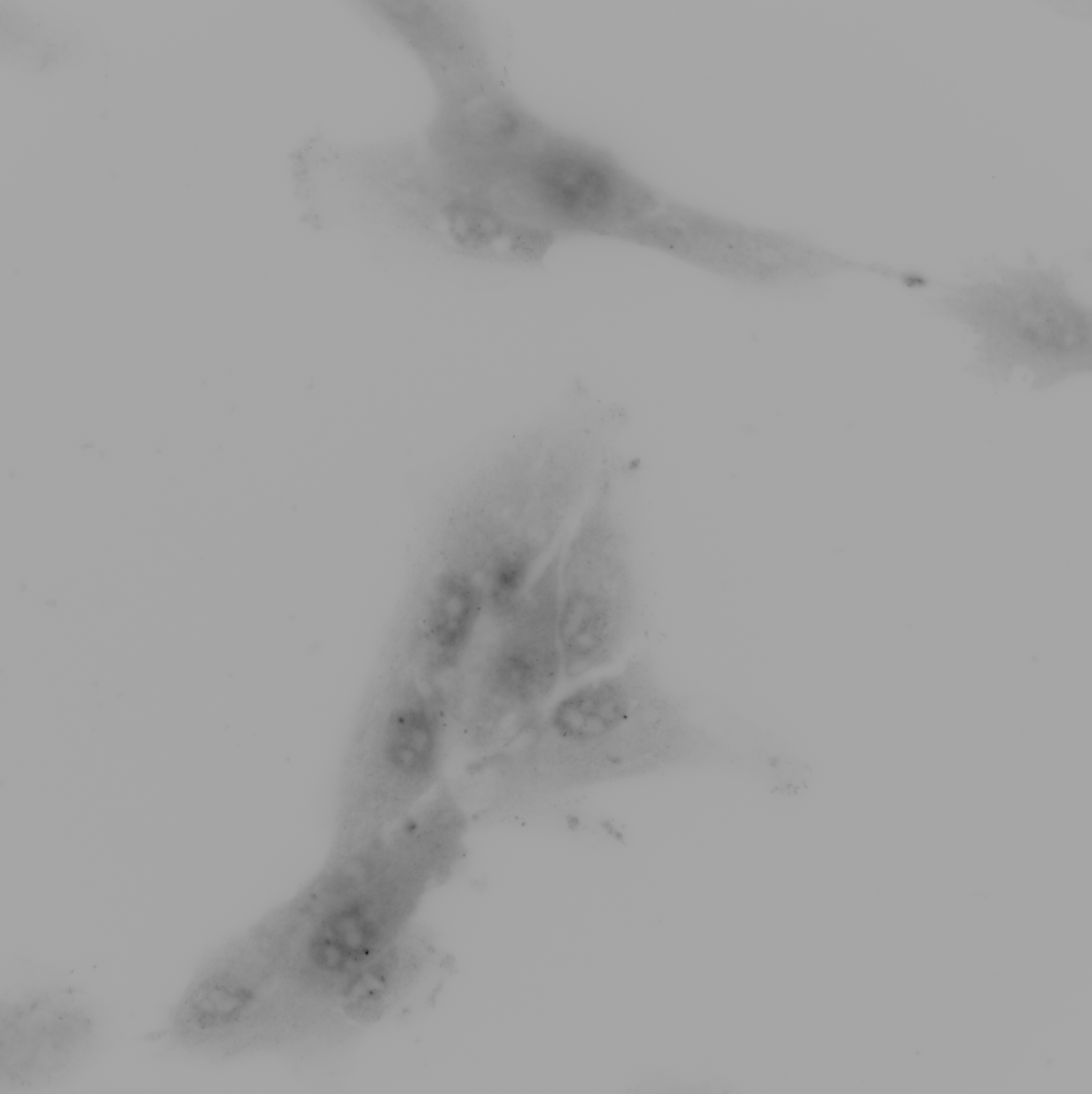

Supplement: Supplementary file 17 — Source data Fig. 5 [file 44318_2024_147_MOESM17_ESM.zip › Figure 5/5C/1CKOclone1_RPE_KIF1CFLmNG_transfection.tiff]

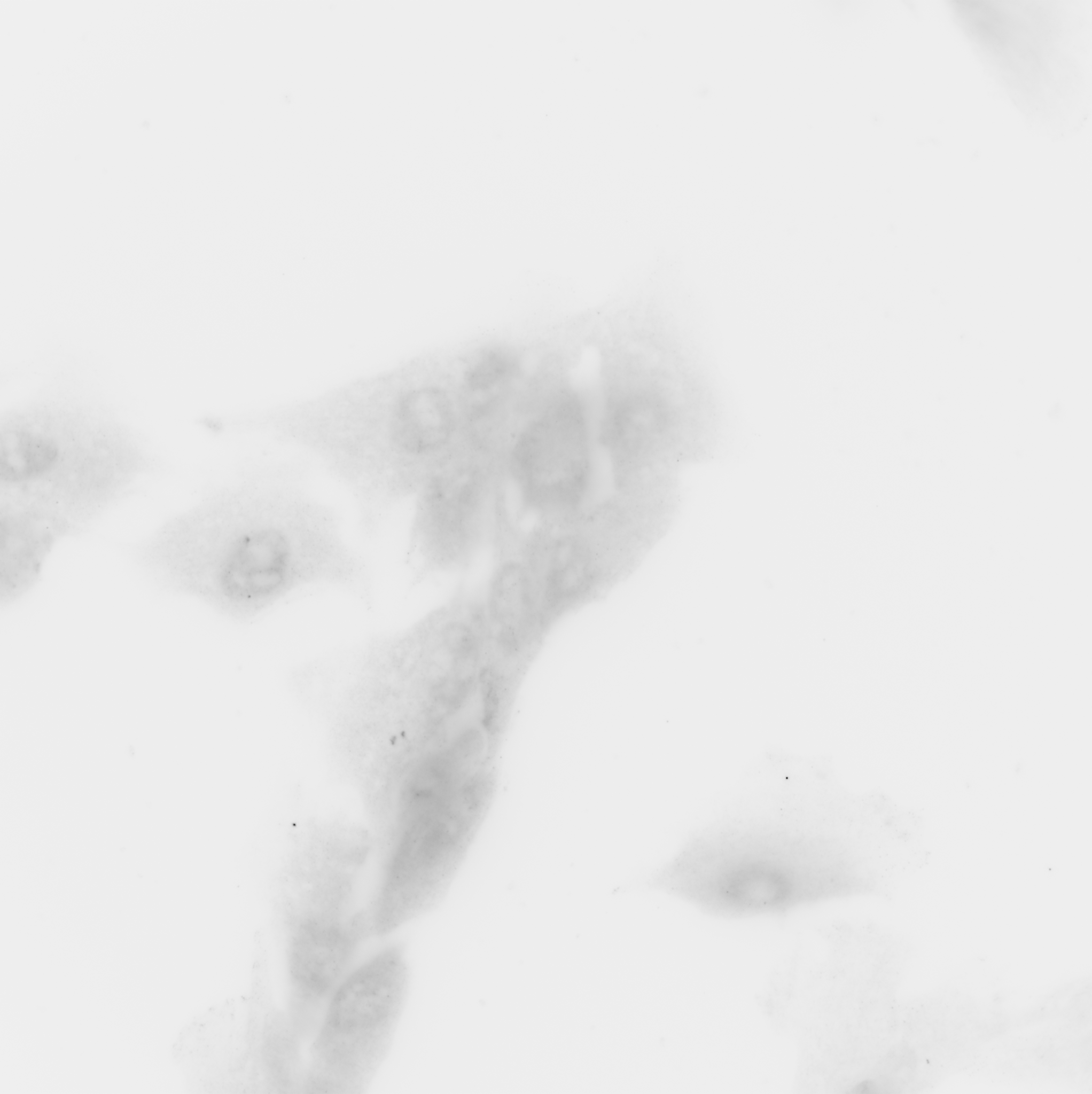

Supplement: Supplementary file 17 — Source data Fig. 5 [file 44318_2024_147_MOESM17_ESM.zip › Figure 5/5D/1CKOclone1_RPE_KIF1CdelIDRmNG_transfection.tiff]

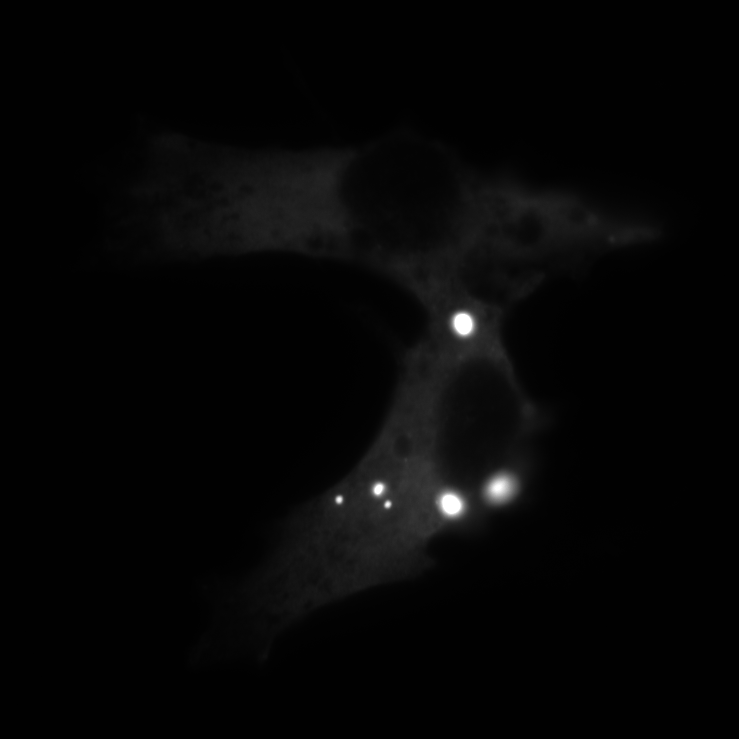

Supplement: Supplementary file 18 — Source data Fig. 6 [file 44318_2024_147_MOESM18_ESM.zip › Figure 6/6E/IDR2a_del_RPE1CKO_80x80um.tif]

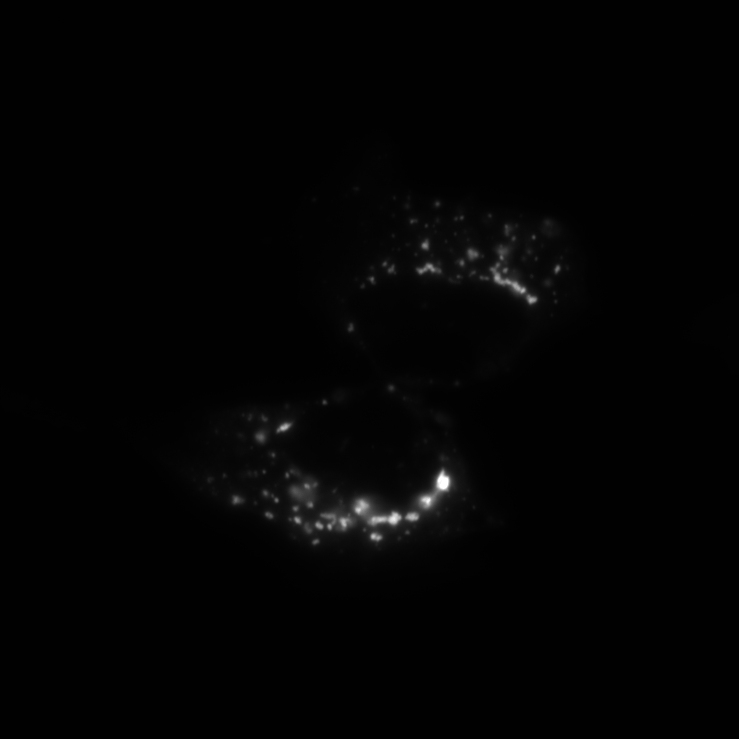

Supplement: Supplementary file 18 — Source data Fig. 6 [file 44318_2024_147_MOESM18_ESM.zip › Figure 6/6E/IDR2b_del_RPE1CKO_80x80um.tif]

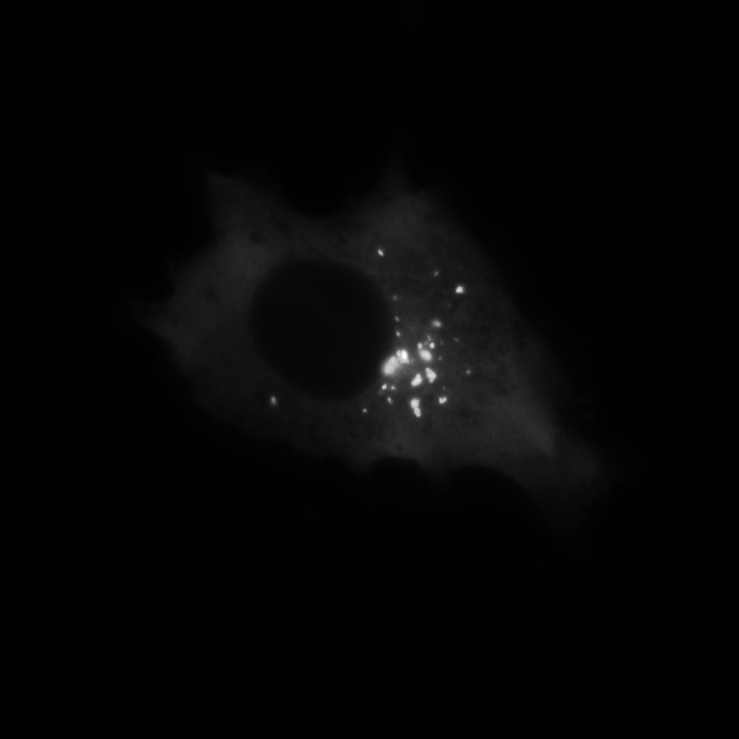

Supplement: Supplementary file 18 — Source data Fig. 6 [file 44318_2024_147_MOESM18_ESM.zip › Figure 6/6D/IDR1_del_RPE1CKO_80x80um.tif]

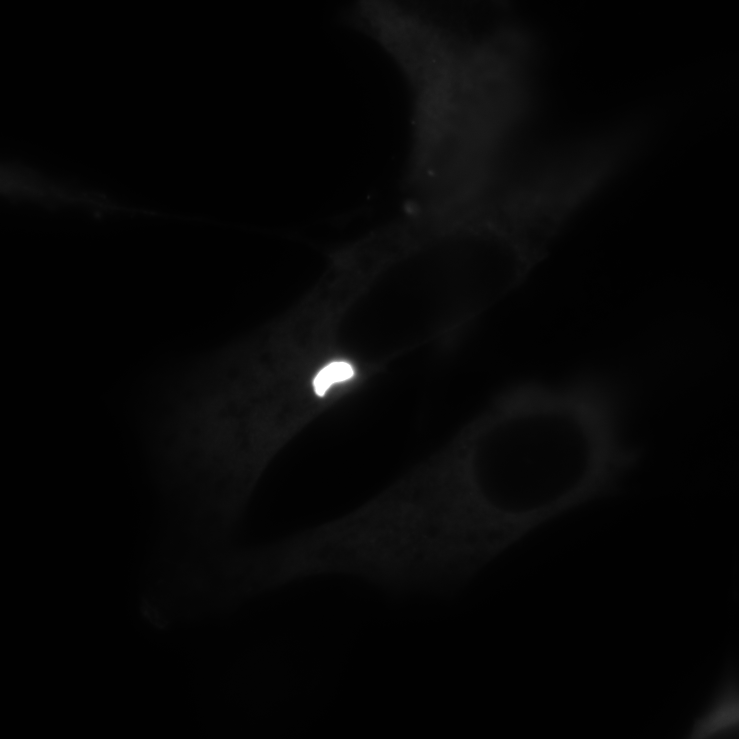

Supplement: Supplementary file 18 — Source data Fig. 6 [file 44318_2024_147_MOESM18_ESM.zip › Figure 6/6D/IDR2_del_RPE1CKO_80x80um.tif]

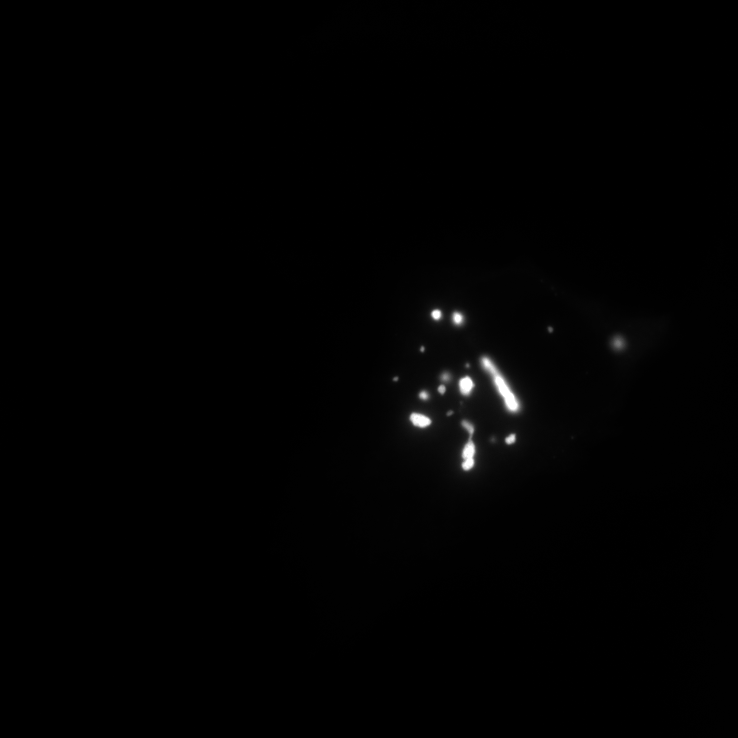

Supplement: Supplementary file 18 — Source data Fig. 6 [file 44318_2024_147_MOESM18_ESM.zip › Figure 6/6D/IDR3_del_RPE1CKO_80x80um.tif]

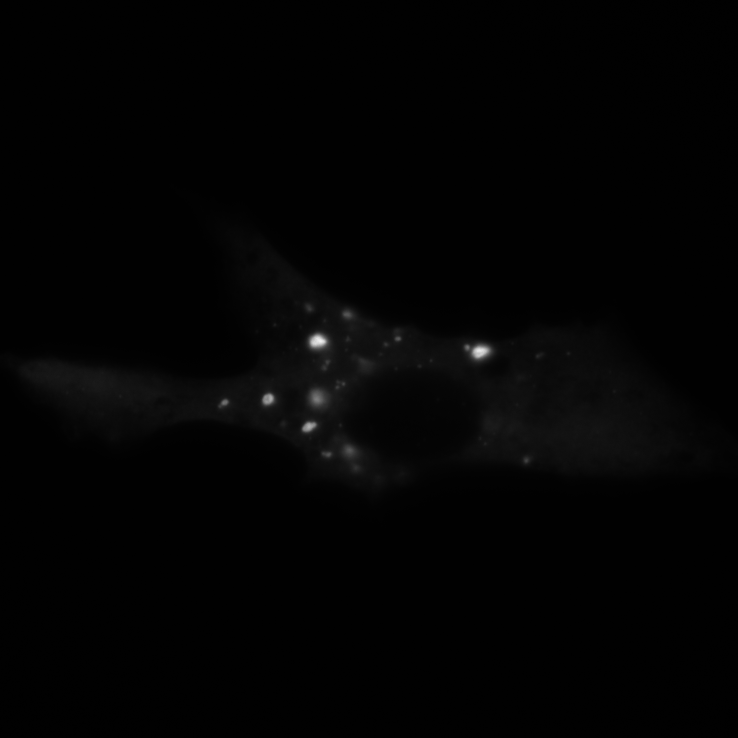

Supplement: Supplementary file 18 — Source data Fig. 6 [file 44318_2024_147_MOESM18_ESM.zip › Figure 6/6C/PLD_del_RPE1CKO_80x80um.tif]

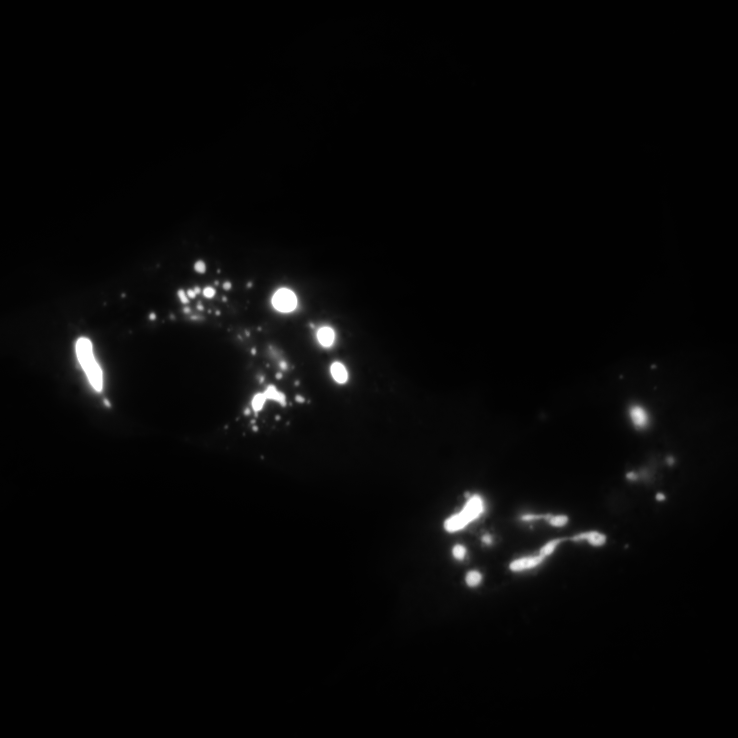

Supplement: Supplementary file 18 — Source data Fig. 6 [file 44318_2024_147_MOESM18_ESM.zip › Figure 6/6C/PLD_mut_RPE1CKO_80x80um.tif]

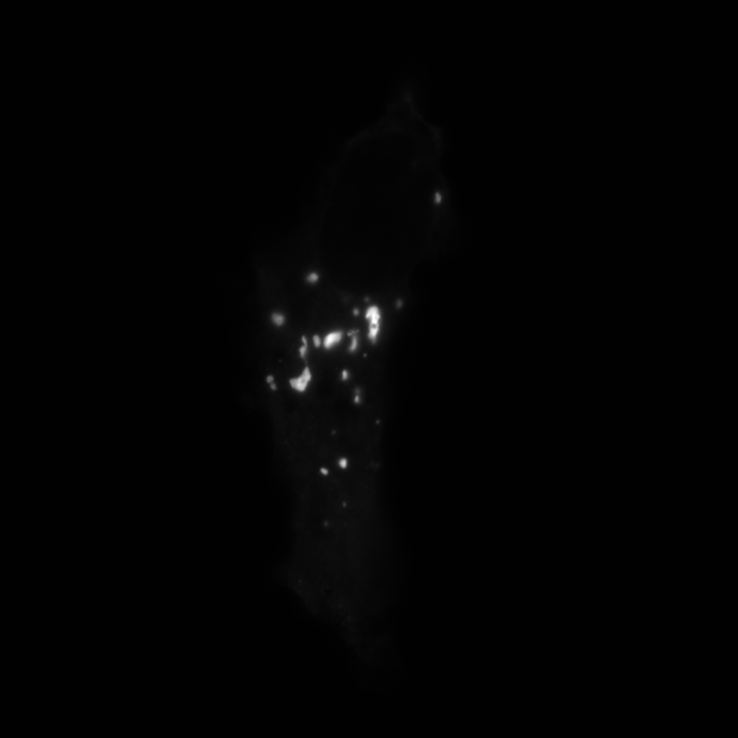

Supplement: Supplementary file 18 — Source data Fig. 6 [file 44318_2024_147_MOESM18_ESM.zip › Figure 6/6C/ST_RPE_1CKOno10B_80x80um.tif]

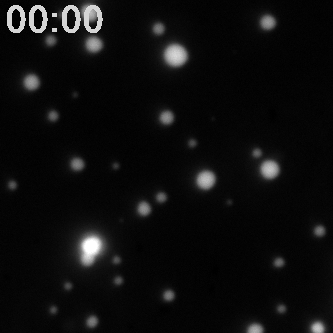

Supplement: Supplementary file 19 — Source data Fig. 7 [file 44318_2024_147_MOESM19_ESM.zip › Figure 7/7D/100mMNaCl_2.72uM_CC4+IDR_20fps.tif]

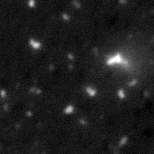

Supplement: Supplementary file 19 — Source data Fig. 7 [file 44318_2024_147_MOESM19_ESM.zip › Figure 7/7B/100mMNaCl_2uM_IDR_10x10um.tif]

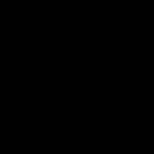

Supplement: Supplementary file 19 — Source data Fig. 7 [file 44318_2024_147_MOESM19_ESM.zip › Figure 7/7B/100mMNaCl_9.4uM_mNG_10x10um.tif]

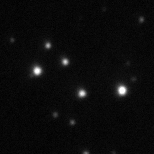

Supplement: Supplementary file 19 — Source data Fig. 7 [file 44318_2024_147_MOESM19_ESM.zip › Figure 7/7B/100mMNaCl_2uM_CC4+IDR_10x10um.tif]

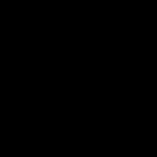

Supplement: Supplementary file 19 — Source data Fig. 7 [file 44318_2024_147_MOESM19_ESM.zip › Figure 7/7E/2.4uMA13_25x25um.tif]

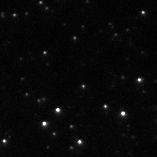

Supplement: Supplementary file 19 — Source data Fig. 7 [file 44318_2024_147_MOESM19_ESM.zip › Figure 7/7E/60nMA13_25x25.tif]

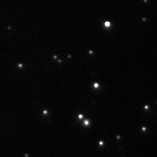

Supplement: Supplementary file 19 — Source data Fig. 7 [file 44318_2024_147_MOESM19_ESM.zip › Figure 7/7E/120nMA13_25x25um.tif]

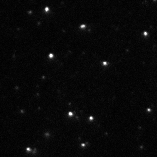

Supplement: Supplementary file 19 — Source data Fig. 7 [file 44318_2024_147_MOESM19_ESM.zip › Figure 7/7E/300nMGU_25x25um.tif]

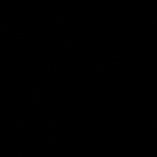

Supplement: Supplementary file 19 — Source data Fig. 7 [file 44318_2024_147_MOESM19_ESM.zip › Figure 7/7E/2.4uMGU_25x25um.tif]

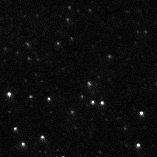

Supplement: Supplementary file 19 — Source data Fig. 7 [file 44318_2024_147_MOESM19_ESM.zip › Figure 7/7E/15nMA13_25x25.tif]

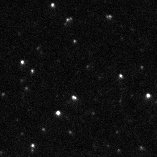

Supplement: Supplementary file 19 — Source data Fig. 7 [file 44318_2024_147_MOESM19_ESM.zip › Figure 7/7E/600nMGU_25x25um.tif]

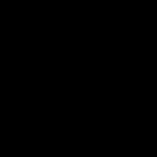

Supplement: Supplementary file 19 — Source data Fig. 7 [file 44318_2024_147_MOESM19_ESM.zip › Figure 7/7E/1.2uMGU_25x25um.tif]

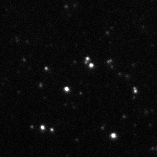

Supplement: Supplementary file 19 — Source data Fig. 7 [file 44318_2024_147_MOESM19_ESM.zip › Figure 7/7E/15nMGU_25x25um.tif]

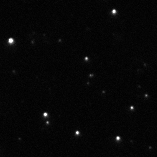

Supplement: Supplementary file 19 — Source data Fig. 7 [file 44318_2024_147_MOESM19_ESM.zip › Figure 7/7E/5nMGU_25x25um.tif]

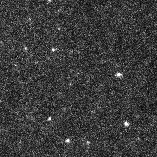

Supplement: Supplementary file 19 — Source data Fig. 7 [file 44318_2024_147_MOESM19_ESM.zip › Figure 7/7E/1.2uMA13_25x25um.tif]

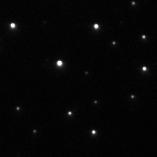

Supplement: Supplementary file 19 — Source data Fig. 7 [file 44318_2024_147_MOESM19_ESM.zip › Figure 7/7E/120nMGU_25x25um.tif]

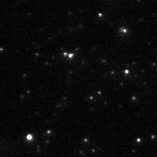

Supplement: Supplementary file 19 — Source data Fig. 7 [file 44318_2024_147_MOESM19_ESM.zip › Figure 7/7E/60nMGU_25x25um.tif]

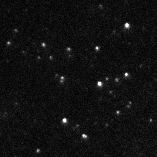

Supplement: Supplementary file 19 — Source data Fig. 7 [file 44318_2024_147_MOESM19_ESM.zip › Figure 7/7E/600nMA13_25x25.tif]

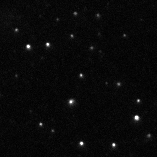

Supplement: Supplementary file 19 — Source data Fig. 7 [file 44318_2024_147_MOESM19_ESM.zip › Figure 7/7E/300nMA13_25x25.tif]

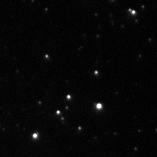

Supplement: Supplementary file 19 — Source data Fig. 7 [file 44318_2024_147_MOESM19_ESM.zip › Figure 7/7E/30nMGU_25x25um.tif]

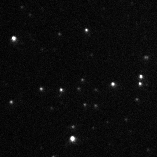

Supplement: Supplementary file 19 — Source data Fig. 7 [file 44318_2024_147_MOESM19_ESM.zip › Figure 7/7E/5nMA13_25x25um.tif]

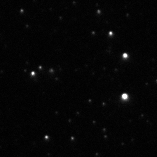

Supplement: Supplementary file 19 — Source data Fig. 7 [file 44318_2024_147_MOESM19_ESM.zip › Figure 7/7E/30nMA13_25x25um.tif]

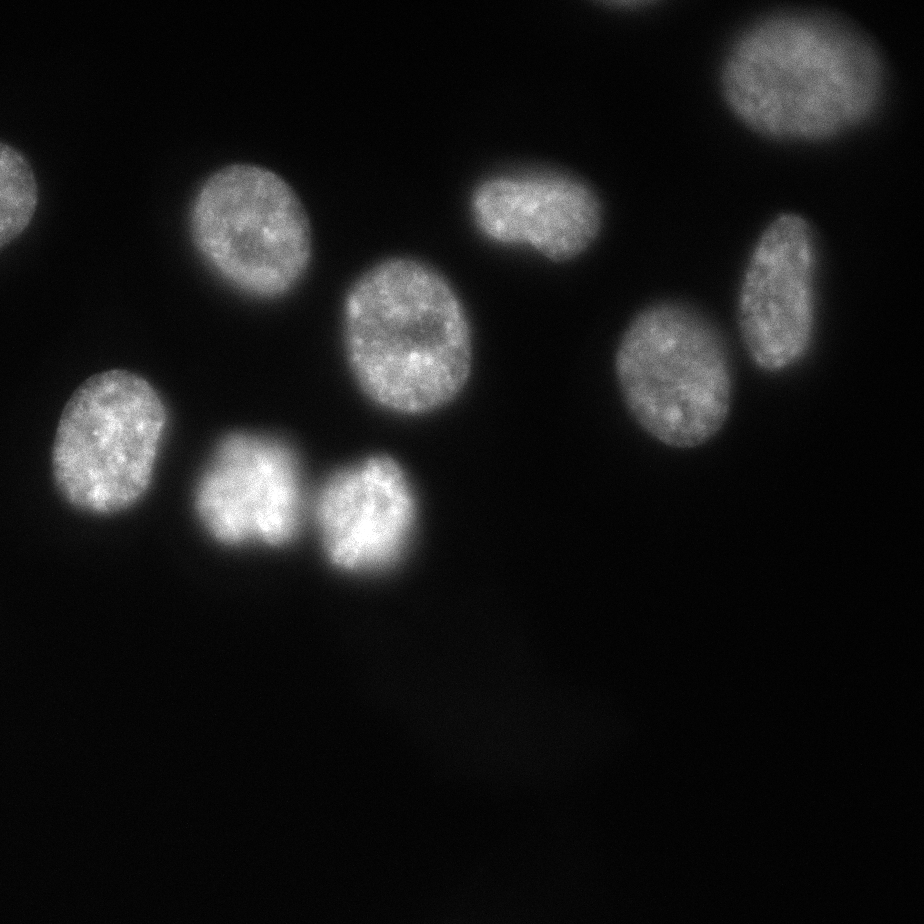

Supplement: Supplementary file 20 — Source data Fig. 8 [file 44318_2024_147_MOESM20_ESM.zip › Figure 8/8D/KIF1CdelIDRmNGstable_KIF1C_IF_100x100um.tif]

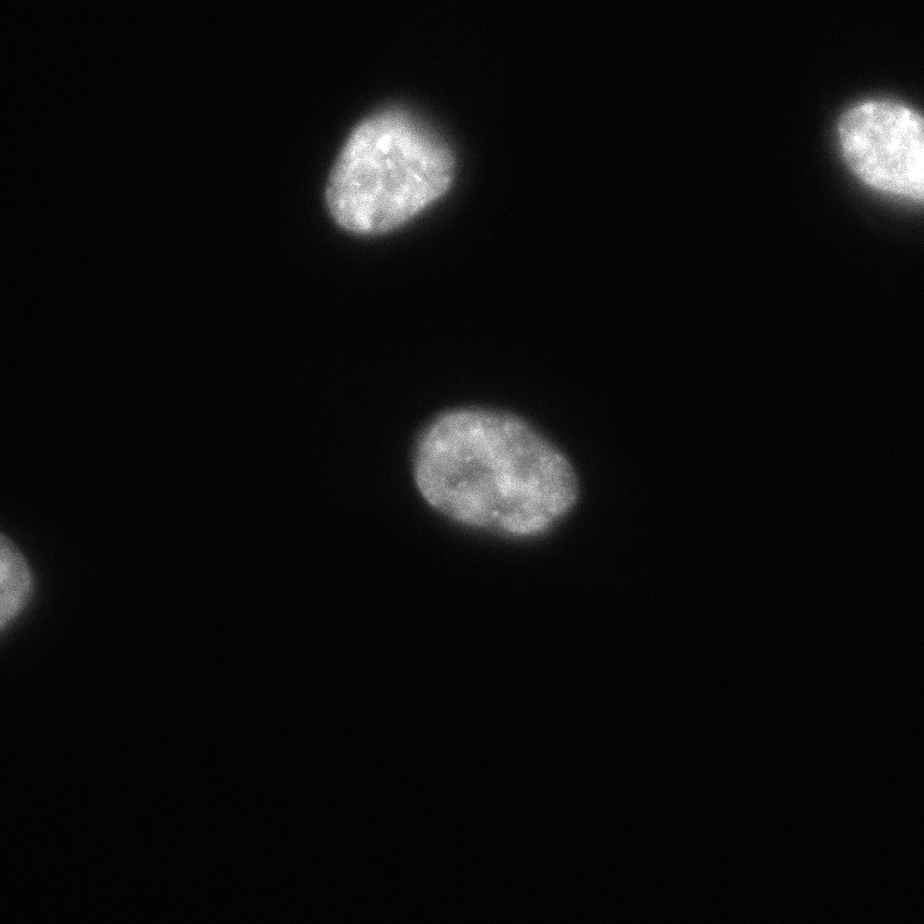

Supplement: Supplementary file 20 — Source data Fig. 8 [file 44318_2024_147_MOESM20_ESM.zip › Figure 8/8D/KIF1CFLmNGstable_KIF1C_IF_100x100um.tif]

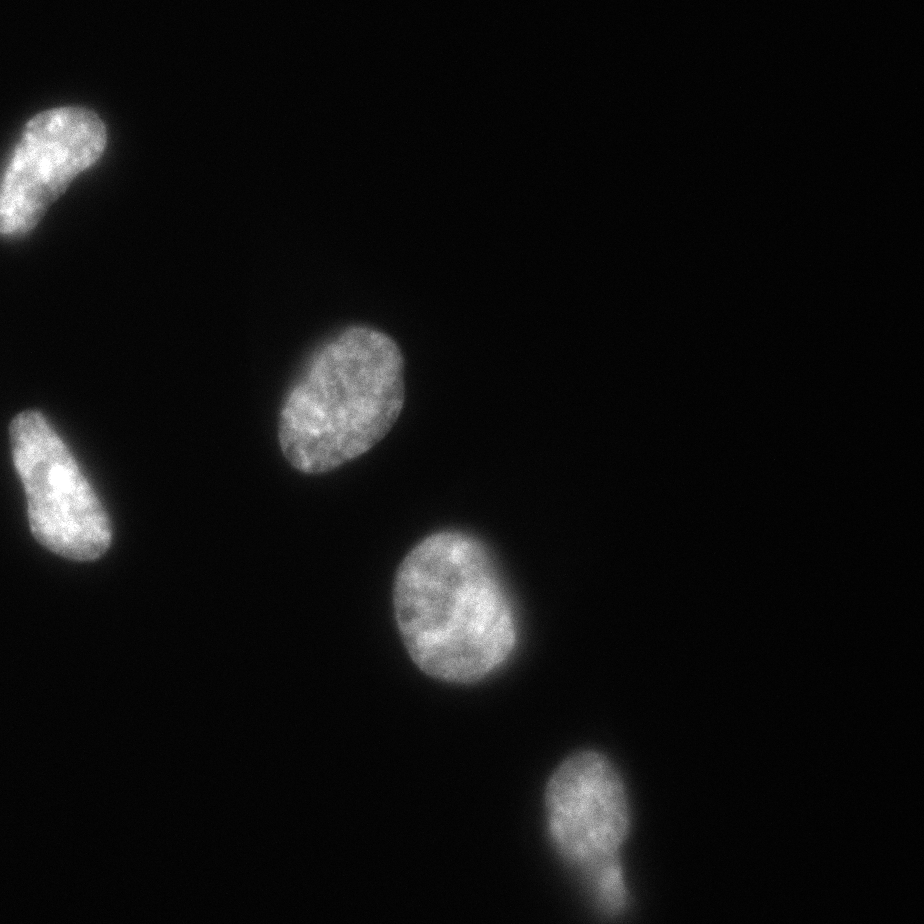

Supplement: Supplementary file 20 — Source data Fig. 8 [file 44318_2024_147_MOESM20_ESM.zip › Figure 8/8B/KIF1CWTRPE_KIF1C_IF_100x100um.tif]

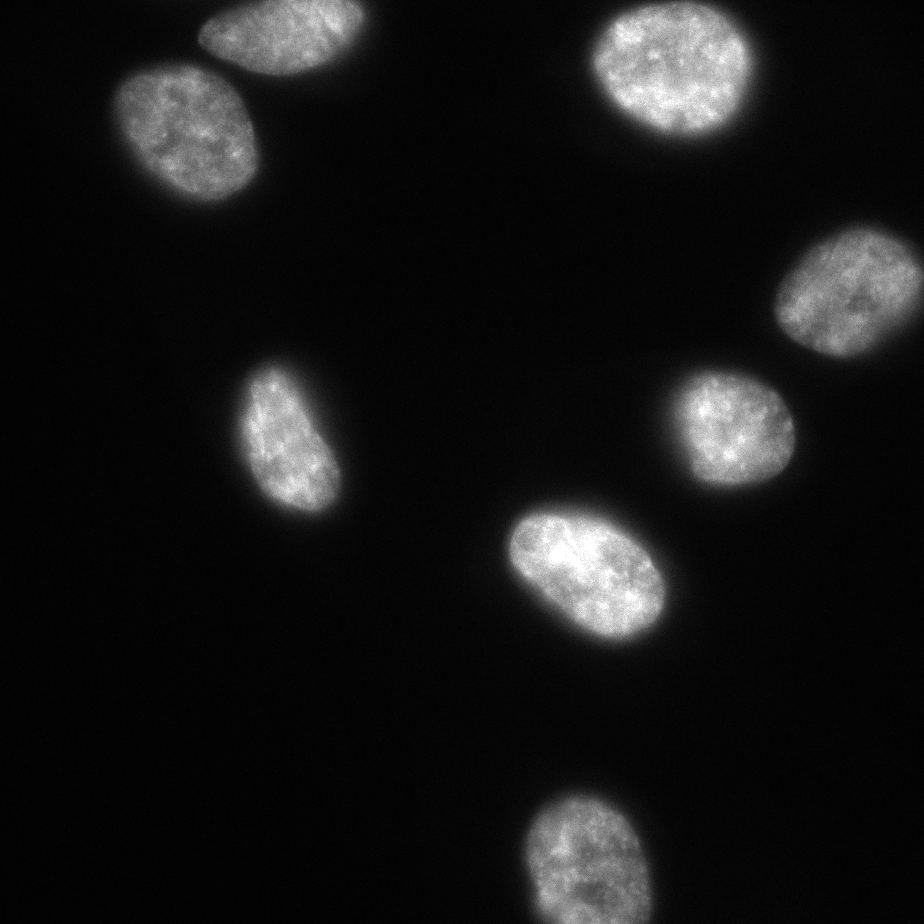

Supplement: Supplementary file 20 — Source data Fig. 8 [file 44318_2024_147_MOESM20_ESM.zip › Figure 8/8B/KIF1CKORPE_KIF1C_IF_100x100um.tif]

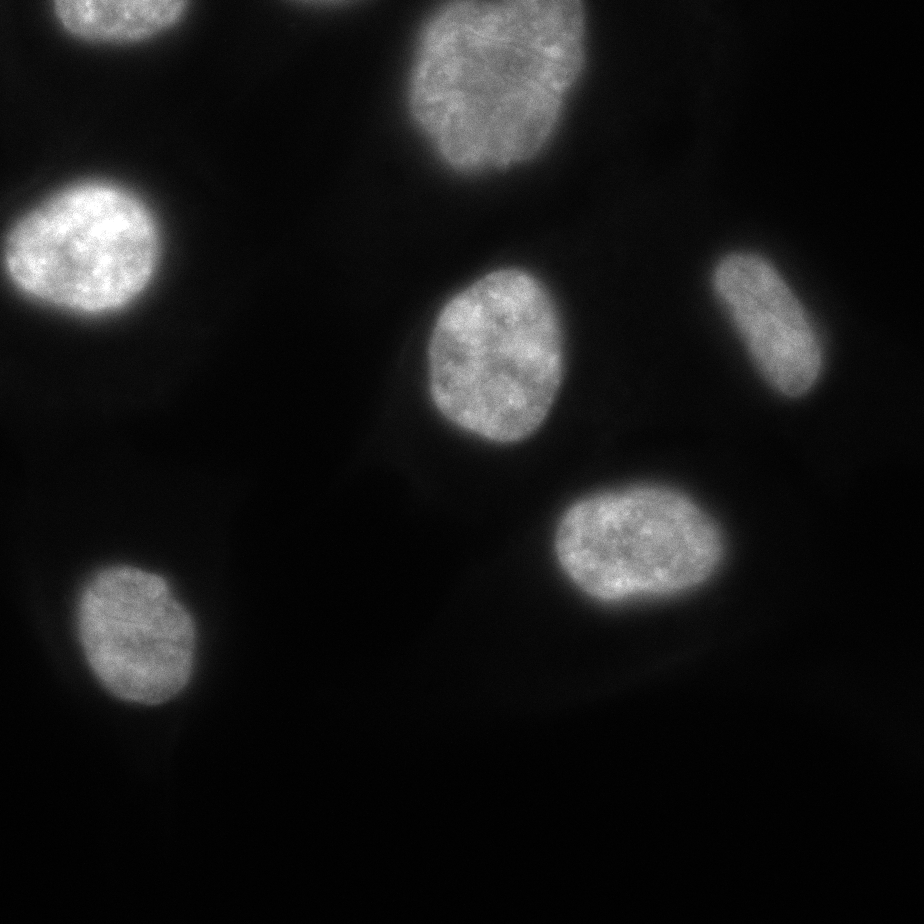

Supplement: Supplementary file 20 — Source data Fig. 8 [file 44318_2024_147_MOESM20_ESM.zip › Figure 8/8E/WTRPE_KIF1CIF_smFISH_RAB13_100x100um.tif]

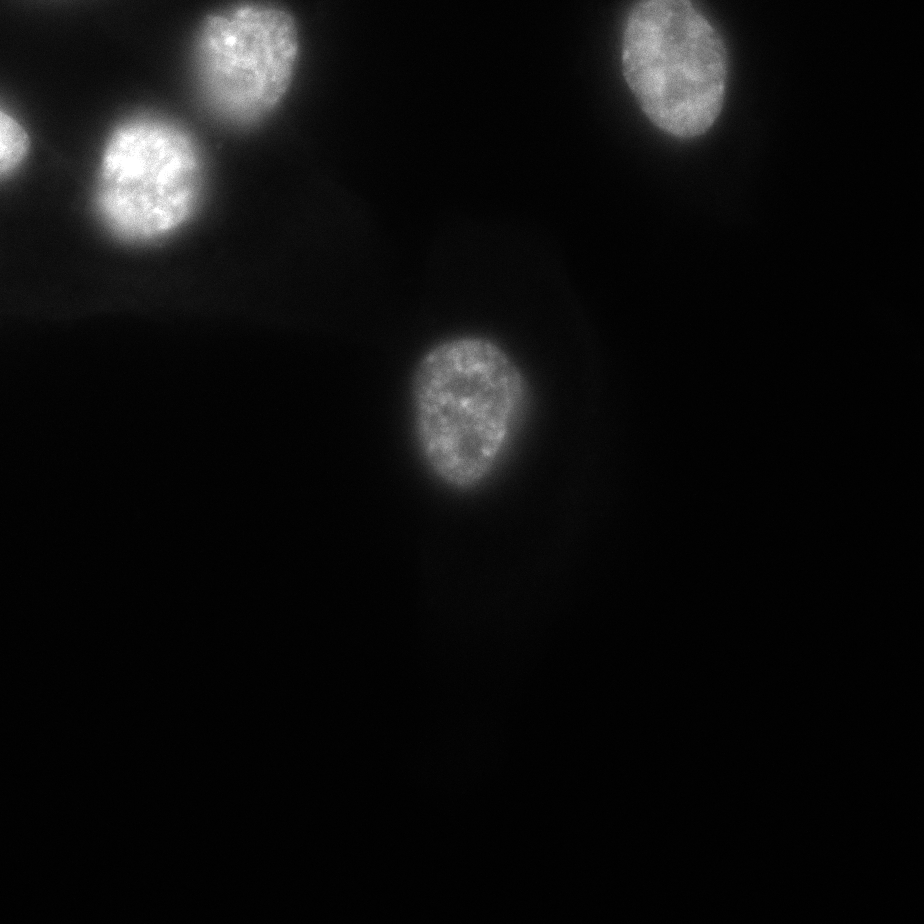

Supplement: Supplementary file 20 — Source data Fig. 8 [file 44318_2024_147_MOESM20_ESM.zip › Figure 8/8E/KIF1CKORPE_KIF1CIF_smFISH_RAB13_100x100um.tif]
